# The cortical critical power law balances energy and information in an optimal fashion

**DOI:** 10.1073/pnas.2418218122

**Published:** 2025-05-23

**Authors:** Tsuyoshi Tatsukawa, Jun-nosuke Teramae

**Affiliations:** ^a^Department of Advanced Mathematical Sciences, Graduate School of Informatics, Kyoto University, Sakyo-ku, Kyoto 606-8502, Japan

**Keywords:** population coding, power law, neural manifolds, Fisher information, computational neuroscience

## Abstract

A recent study has suggested that the stimulus responses of cortical neural populations follow a critical power law. More precisely, the power spectrum of the covariance matrix of neural responses follows a power law with an exponent indicating that the neural manifold lies on the edge of differentiability. This criticality is hypothesized to balance expressivity and robustness in neural encoding, as population responses on a nondifferential fractal manifold are thought to be overly sensitive to perturbations. However, contrary to this hypothesis, we prove that neural coding is far more robust than previously assumed. We develop a theoretical framework that provides an analytical expression for the Fisher information of population coding under the small noise assumption. Our results reveal that, due to its intrinsic high dimensionality, population coding maintains reliability even on a nondifferentiable fractal manifold, despite its sensitivity to perturbations. Furthermore, the theory reveals that the trade-off between energetic cost and information makes the critical power-law coding the optimal neural encoding of sensory information for a wide range of conditions. In this derivation, we highlight the essential role of a neural correlation, known as differential correlation, in power-law population coding. By uncovering the nontrivial nature of high-dimensional information coding, this work deepens our understanding of criticality and power laws in both biological and artificial neural computation.

## Significance

How neural populations in the brain represent sensory information is one of the central questions in neuroscience. To ensure robustness against noise, it is widely believed that the neural representation must avoid the so-called fractal state, where the population response becomes unsmooth and overly sensitive to input perturbations. However, by analyzing the Fisher information, we prove that population coding is far more robust than previously thought. Counterintuitively, due to its intrinsic high dimensionality, population representation does not degrade even in highly sensitive regimes. With this result, we show that the trade-off between energy consumption and coding efficiency results in the critical power law, a recently discovered remarkable feature of population responses, being the optimal population encoding of sensory information.

How the activity of a population of neurons in the brain represents, or encodes, external signals such as visual images presented to animals has long been a central question in both neuroscience and machine learning (1–16). While the actual structure of the coding has been largely unknown, two seemingly contradictory hypotheses have been proposed. One is the efficient coding hypothesis, which suggests that population coding should be high-dimensional and sparse to reduce input stimulus correlations, thereby facilitating easier decoding (1, 2, 4, 16). The other is the low-dimensional subspace hypothesis. This hypothesis proposes that population activities are confined to low-dimensional subspaces, or manifolds, to enhance robustness against noise (3, 5, 7, 9, 14, 15).

**Fig. 1.**
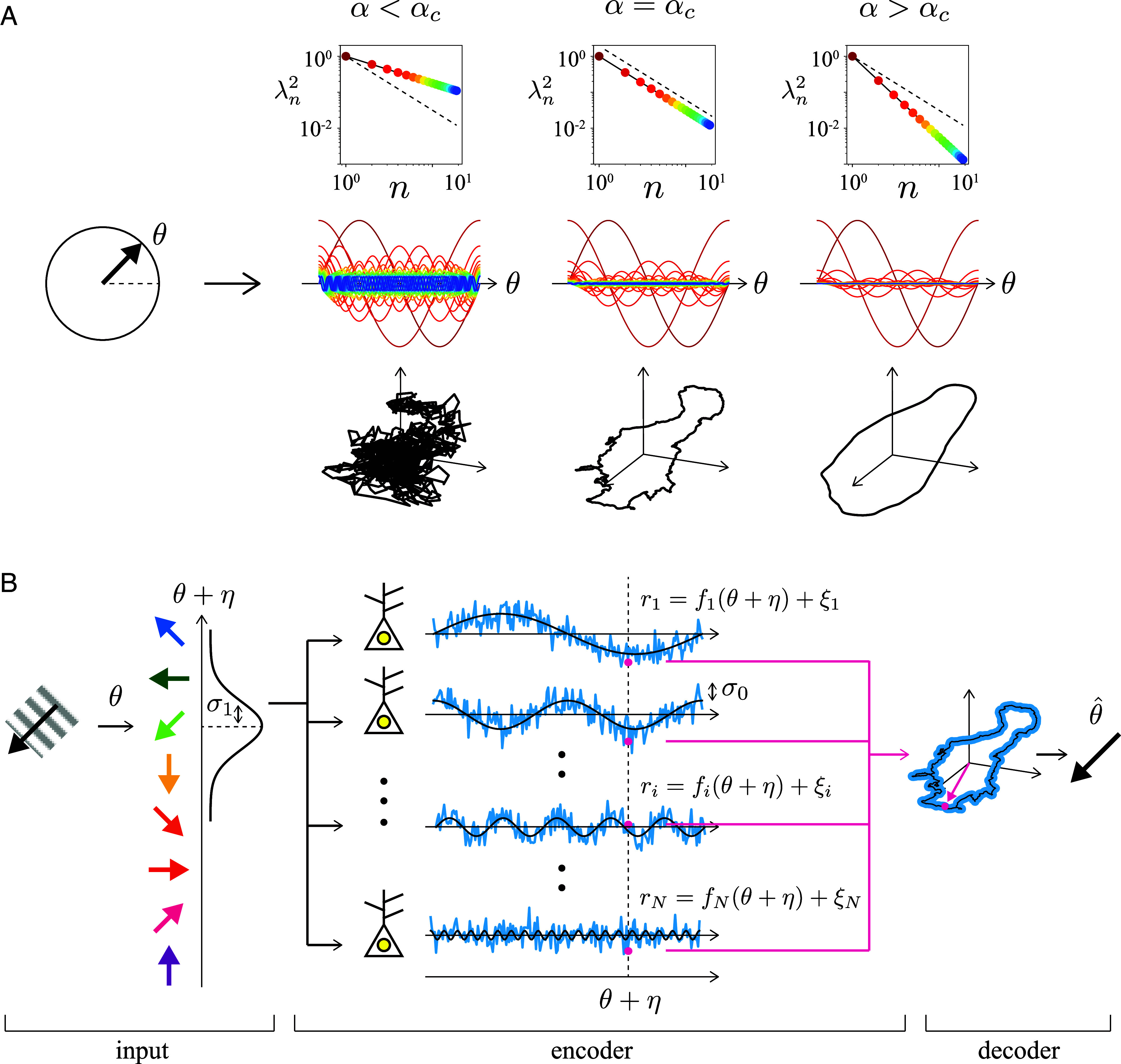
Power-law responses of cortical neurons and an analytically tractable coding model with the power-law responses. (*A*) The variance of the nth principal component, i.e., the square of the eigenspectrum of the covariance matrix, of population activities of cortical neurons in response to input stimuli follows the power law with $\lambda_{n}^{2}\propto k^{-\alpha }$ (upper rows). If the exponent α is smaller than the critical value $\alpha_{c}$, the neural manifold of the stimulus representation will be high-dimensional and nondifferentiable (left column). In contrast, the manifold will be low-dimensional and smooth if the value of α is larger than $\alpha_{c}$ (right column). In this sense, the experimentally observed exponent close to $\alpha_{c}$ is critical (middle column). Panels of the middle row show examples of receptive fields of neurons (different colors corresponding to different neurons) where the input stimulus θ is a one-dimensional periodic signal, and neural responses obey a power law each. Lower panels depict three-dimensional projections of the neural manifolds, that is, projections of neural responses shown in the middle rows to randomly chosen three directions when the input θ varies from 0 to 2π. (*B*) Outline of the encoding model with the power-law stimulus responses. Input stimulus θ (furthest to the left) added an intrinsic noise η with strength $\sigma_{1}$ is given to the population of neurons. Each neuron responds to the input with the activity following its receptive field whose amplitude obeys the power law $f_{i}\left(\theta +\eta \right)$, which is further added random noise ξ of the strength $\sigma_{0}$ which represents the randomness of the neural activity. Thus, the population activity is given as a perturbed point on the neural manifold. Finally, the output neuron, or the estimator (furthest to the right), decodes the population activity to obtain the estimated value $\hat{\theta }$ of the input.

Recent advancements in recording techniques suggest the possible structure of population coding in the brain (8). These findings indicate that cortical coding is situated at a midpoint between two competing hypotheses. Simultaneous recordings of neurons in the primary visual cortex in vivo demonstrate that the eigenspectrum of the covariance matrix of neural activity, marginalized over input stimuli, follows a power law. In particular, the variance of the nth dimension of population activity decreases as a power of n, approximately in ascending order of its wavenumber or frequency response to the input (Fig. 1*A*). The exponent of this power-law decay agrees well with $\alpha_{c}=1+2/D$, independent of input statistics, where D represents the input dimension. For natural images, which have almost infinite dimensions, the power spectrum decays as 1/n. The efficient coding hypothesis predicts a nearly flat power spectrum, while the low-dimensional subspace hypothesis suggests rapid decay. The observed power-law decay, therefore, supports the idea that the brain operates at an intermediate point between these two extremes.

Stringer et al. (8) demonstrated that the exponent $\alpha_{c}$ is a critical value that defines the border of differentiability for the coding manifold. They mathematically proved that, in the limit of a large number of neurons, if the map from the input space to the neural activity space is differentiable, that is, if the neural manifold is differentiable, then the power spectrum must asymptotically decay faster than the critical exponent $\alpha_{c}$. Conversely, if the decay is slower than $\alpha_{c}$, the map becomes nondifferentiable, resulting in a fractal-like neural response. In this case, the coding becomes too sensitive to input perturbations, as even infinitesimally close inputs elicit significantly different neural activities. It is therefore conjectured that the brain optimizes the sensitivity of its code to inputs while maintaining smoothness, achieving a balance between expressiveness and robustness in its code. The similar critical sensitivity to perturbations also plays an important role in studies of machine learning (17–24).

It is truly fascinating that the brain’s signal encoding is in a critical state (25–28). However, quantitative evaluation of the performance of the power-law coding remains elusive probably due to the lack of the framework to theoretically describe the coding performance of the power-law. For example, it remains unclear how the nondifferentiability of the neural manifold indeed affects the coding performance, and why the critical state should be used by the brain as an encoder.

In this study, we address this problem by applying the framework of statistical parameter estimation to power-law coding, with properly accounting for possible noise sources (29–35). In this framework, optimal neural coding is defined as a code that allows decoders to estimate the encoded signals with minimal error. The effectiveness of the stochastic coding is measured by its Fisher information, as the inverse of the Fisher information provides the lower bound of the estimation error variance for unbiased decoders (Cramér–Rao bound) and the asymptotic variance of the error for maximum likelihood decoders in the limit of a large number of observations (36).

We will derive analytical expressions for the Fisher information of the neural power-law coding and prove that: i) the exponent $\alpha_{c}$ indeed gives the critical point, as Fisher information becomes discontinuous only at this exponent in the limit of a large number of neurons. Unexpectedly, however, ii) the Fisher information is constant rather than decreasing even for the exponent smaller than the critical value, where the coding manifold is nondifferentiable. Thus, contrary to the previous conjecture, the critical power law is not always the best coding in the information-theoretic sense. Why, then, does the brain use the critical coding? We will show that iii) introducing an energetic cost ensures the optimality of the critical coding. By computing the Fisher information, we will also prove a remarkable relationship between the variance and the susceptibility of neural activity and highlight the essential role of stimulus correlations, more precisely differential correlations for the sensory information encoding (34, 35). To validate the theoretical predictions, we will construct a maximum likelihood decoder for the power-law coding by explicitly integrating the conditional probability distribution of the encoded input signal. This allows us to measure the variance of its estimation errors and to numerically evaluate the Fisher information of the power-law coding.

## Results

### Statistical Estimation with the Power-Law Population Coding.

Following Stringer’s pioneering work (8), let us start from the simplest case where a population of neurons encodes a one-dimensional periodic scalar angular variable, θ, such as the orientation of a line segment presented in animal’s visual field. Therefore, D=1 (Fig. 1*B*) here. Generalizations to higher input dimensions will be given later. This coding can be affected by two different noise sources; one is the input noise that is put directly to the input stimulus θ and the other is the neural noise being independently put on each neural activity evoked by the input stimulus. We model the input noise η and the neural noise for the ith neuron $\xi_{i}$ using the independent Gaussian random variables satisfying that $\left\langle \xi_{i}\right\rangle =\left\langle \eta \right\rangle =\left\langle \eta \xi_{i}\right\rangle =0$, $\left\langle \eta^{2}\right\rangle =\sigma_{1}^{2}$, and $\left\langle \xi_{i}\xi_{j}\right\rangle =\sigma_{0}^{2}\delta_{\mathrm{ij}}$, where $\sigma_{1}$ and $\sigma_{0}$ are the strengths of the input and neural noise, respectively. Experimental findings have shown that the variance of the principal components, i.e., the eigenspectrum of the covariance matrix of neural activities, decays with the power law in ascending order of frequency. Based on this observation, we express the neural activity of the ith neuron (i=1,…,2N) as an expansion over the orthonormal Fourier basis of a scalar periodic function:

$$\begin{array}{c} r_{i}\left(\theta \right)=\sum_{j=1}^{2N}a_{\mathrm{ij}}f_{j}\left(\theta +\eta \right)+\xi_{i}, \end{array}$$

where

$$\begin{array}{c} f_{j}\left(\theta \right)=\left\{\begin{array}{cc} cn^{-\alpha /2}\cos n\theta & \left(j=2n-1\right)\\ cn^{-\alpha /2}\sin n\theta & \left(j=2n\right) \end{array}\right.. \end{array}$$

Here, $A=[a_{\mathrm{ij}}]$ is an orthogonal matrix that rotates the axes of the basis function to the axes of the neuron space, and c is a scaling factor that determines the magnitude of the activity of the neurons. However, by using a basis change, we can set A to be the identity matrix and c=1 without loss of generality, which gives the stochastic coding model as,

[1] $$\begin{array}{c} r_{i}\left(\theta \right)=\left\{\begin{array}{cc} n^{-\alpha /2}\cos n(\theta +\eta )+\xi_{i} & \left(i=2n-1\right)\\ n^{-\alpha /2}\sin n(\theta +\eta )+\xi_{i} & \left(i=2n\right) \end{array}\right.. \end{array}$$

Note that the axis change does not affect the strength of the neural noise since the noise is isotropic in the neural space.

The Fisher information of the stochastic coding is defined as the variance of the score function, the derivative of the loglikelihood function with respect to the input θ, or equivalently, the negative mean of the second derivative of the loglikelihood function (30, 36)

[2] $$\begin{array}{c} I\left(\theta \right)={\langle {\left(\frac{\partial \;\log\;p(r;\;\theta )}{\partial \theta }\right)}^{2}\rangle }_{r}={\langle -\frac{\partial^{2}\;\log\;p(r;\;\theta )}{\partial \theta^{2}}\rangle }_{r}. \end{array}$$

Here, p(r;θ) denotes the probability distribution of the neuronal activity $r={\left(r_{1},\ldots ,r_{2N}\right)}^{⊤}$ responding to the input θ. A variable transformation from the Gaussian variables to the neural activities gives the explicit form of the distribution function as a convolutional integral of Gaussian functions,

[3] $$\begin{array}{cc} p(r;\;\theta ) & =\frac{1}{{\left(2\pi \sigma_{1}^{2}\right)}^{\frac{1}{2}}{\left(2\pi \sigma_{0}^{2}\right)}^{N}}\int d\phi \exp[-\frac{1}{2\sigma_{1}^{2}}{\left(\phi -\theta \right)}^{2}\\ & \;-\frac{1}{2\sigma_{0}^{2}}\sum_{n=1}^{N}({\left(x_{n}-n^{-\alpha /2}\cos n\phi \right)}^{2}\\ & \;+{\left(y_{n}-n^{-\alpha /2}\sin n\phi \right)}^{2})], \end{array}$$

where we denote $r_{2n-1}=x_{n}$ and $r_{2n}=y_{n}$ for simplicity (see *SI Appendix*, section 1 for details).

### Susceptibility–Variance Relationship and the Fisher Information of the Power-Law Coding.

To derive an analytical expression for the Fisher information, let us assume that the noise strengths $\sigma_{0}$ and $\sigma_{1}$ are sufficiently small so that the probability distribution can be approximated by the multivariate Gaussian distribution,

[4] $$\begin{array}{c} p\left(r;\;\theta \right)\approx \frac{1}{\sqrt{{\left(2\pi \right)}^{2N}\left|\Sigma \right|}}\exp\left(-{\left(r-m\right)}^{⊤}\Sigma^{-1}\left(r-m\right)\right), \end{array}$$

where m and Σ are the mean and the covariance matrix of the neural activity r for given θ, respectively, and $x^{⊤}$ denotes the transpose of x.

**Fig. 2.**
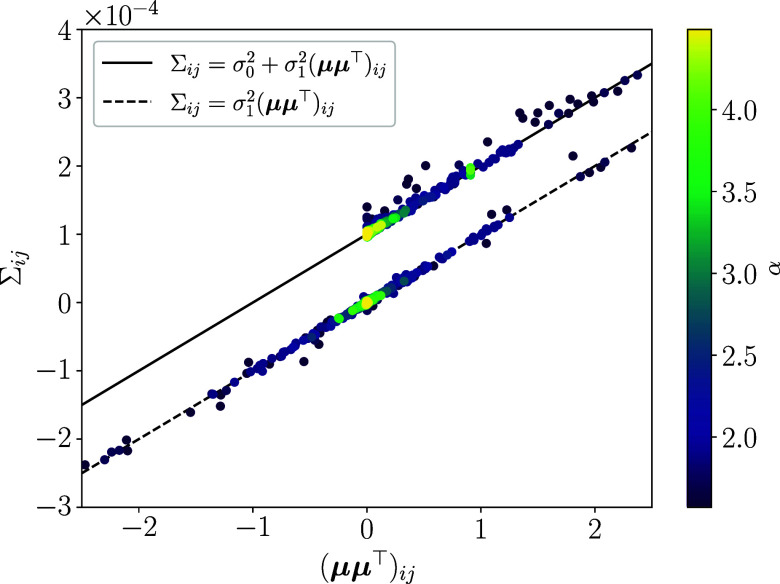
Susceptibility–variance relationship of the neural response. Each point represents an element of the covariance matrix $\Sigma_{\mathrm{ij}}$ as a function of the corresponding element of the outer product of the susceptibility of the neural response to the input signal ${\left(\mu \mu^{⊤}\right)}_{\mathrm{ij}}$. The color of the points indicates the value of α. As predicted by Eq. **5**, the covariance is proportional to the outer product for off-diagonal elements. They, however, are shifted by $\sigma_{0}^{2}$ to the vertical direction for diagonal elements due to the identity matrix of the first term of the equation. The thick line is $\Sigma_{\mathrm{ij}}=\sigma_{0}^{2}+\sigma_{1}^{2}{\left(\mu \mu^{⊤}\right)}_{\mathrm{ij}}$, and the dashed line is $\Sigma_{\mathrm{ij}}=\sigma_{1}^{2}{\left(\mu \mu^{⊤}\right)}_{\mathrm{ij}}$. We used $\sigma_{1}=0.01$, $\sigma_{0}=0.01$ and N=100 for the plot.

Now let us denote the derivative of the mean with respect to the input θ as μ=∂m/∂θ, namely, μ is the susceptibility of the mean neural activity to the input signal. Then, we can derive a remarkable relationship between the covariance matrix Σ and the susceptibility μ:

[5] $$\begin{array}{c} \Sigma =\sigma_{0}^{2}I+\sigma_{1}^{2}\mu \mu^{⊤}, \end{array}$$

where I is the identity matrix (*SI Appendix*, section 2A for details). The second term of the covariance matrix is referred to as differential correlation, which has been extensively studied to uncover the underlying mechanisms shaping the representation of information in neural coding (34, 35). To confirm this identity, we directly measure the covariance matrix and the input susceptibility for the population activity of neurons given by Eq. **1** for various realizations of the exponent α, input noise η, and neuron noise ξ. Fig. 2 is a scatter plot of elements of the covariance matrix $\Sigma_{\mathrm{ij}}$ versus the corresponding outer products of the susceptibility vectors ${\left(\mu \mu^{⊤}\right)}_{\mathrm{ij}}$. We see that regardless of the parameter realizations, both diagonal (points along the solid line) and off-diagonal elements (points along the dashed line) well agree with the theoretical prediction.

The above relation also gives the impressive result that the susceptibility is an eigenvector of the covariance matrix, whose eigenvalue is given by using the generalized harmonic function $H_{N}\left(x\right)=\sum_{n=1}^{N}n^{-x}$, which converges to the Riemann zeta function $\zeta \left(x\right)=\sum_{n=1}^{\infty }n^{-x}$ in the limit of large numbers of neurons N→∞ (*SI Appendix*, section 2B for details),

[6] $$\begin{array}{c} \begin{array}{cc} \Sigma \mu & =\lambda \mu \\ \lambda & =\sigma_{0}^{2}+\sigma_{1}^{2}H_{N}\left(\alpha -2\right)\\ & \to \sigma_{0}^{2}+\sigma_{1}^{2}\zeta \left(\alpha -2\right). \end{array} \end{array}$$

Putting Eqs. **4** and **5** to Eq. **2** and using Eq. **6**, we have the Fisher information of the power-law coding as

[7] $$\begin{array}{c} I\left(\theta \right)=\frac{H_{N}\left(\alpha -2\right)}{\sigma_{0}^{2}+\sigma_{1}^{2}H_{N}\left(\alpha -2\right)}, \end{array}$$

which converges to

[8] $$\begin{array}{c} I_{1}\left(\alpha \right):=\frac{\zeta (\alpha -2)}{\sigma_{0}^{2}+\sigma_{1}^{2}\zeta \left(\alpha -2\right)}, \end{array}$$

in the limit of a large number of neurons.

The generalization to D>1, where neurons encode a higher-dimensional input, is almost straightforward (*SI Appendix*, section 3 for details). The Fisher information for multivariate input signal θ is defined in matrix form,

$$\begin{array}{c} I_{\mathrm{ij}}\left(\theta \right)={\langle -\frac{\partial^{2}\;\log\;p\left(r;\;\theta \right)}{\partial \theta_{i}\partial \theta_{j}}\rangle }_{r}. \end{array}$$

If the Gaussian noise with strength $\sigma_{i}$ independently affects the ith input component $\theta_{i}$, then, using the same argument as above, we can show that the Fisher information matrix becomes diagonal. In the limit of a large number of neurons, the ith diagonal component, which evaluates the performance of the power-law coding for the ith input signal $\theta_{i}$, converges to:

[9] $$\begin{array}{c} I_{\mathrm{ii}}\left(\theta \right)\to I_{D}\left(\alpha \right):=\frac{\zeta \left(\alpha -2/D\right)}{\sigma_{0}^{2}DV_{D}^{2/D}/4+\sigma_{i}^{2}\zeta \left(\alpha -2/D\right)}, \end{array}$$

where $V_{D}=\pi^{D/2}/\Gamma \left(D/2+1\right)$ is the volume of the unit D-ball. This reproduces Eq. **8** as a special case. Then, in the limit of a large input dimension, D→∞, it converges to

[10] $$\begin{array}{c} I_{\infty }\left(\alpha \right)=\frac{\zeta (\alpha )}{e\pi \sigma_{0}^{2}/2+\sigma_{i}^{2}\zeta \left(\alpha \right)} \end{array}$$

because $\Gamma \left(z+1\right)\approx \sqrt{2\pi z}{\left(z/e\right)}^{z}$ for large z.

**Fig. 3.**
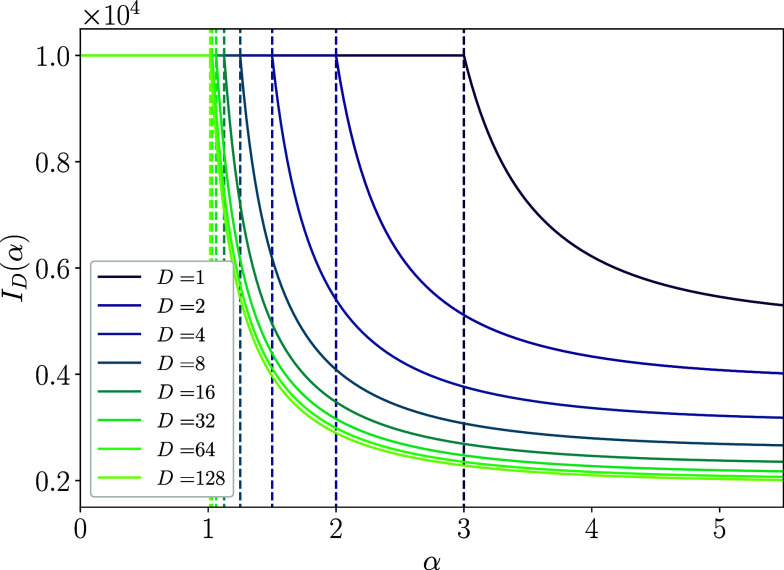
Analytically derived Fisher information $I_{D}$ as a function of the power-law exponent α for various values of the input dimension D. Vertical dashed lines indicate the critical values of the exponent $\alpha_{c}=1+2/D$. For all D, the information rapidly decreases as α increases for $\alpha >\alpha_{c}$. However, counterintuitively, they keep a constant value for $\alpha <\alpha_{c}$ even though the neural representation is intrinsically nondifferentiable, the neural manifold is fractal, and thus, the population response is highly sensitive to the perturbation in the regime. Here, we used $\sigma_{0}=\sigma_{1}=0.01$.

Fig. 3 shows the derived analytical expression of the Fisher information Eq. **9** as functions of the power-law exponent α for various values of the input dimension D. One can see three significant features from the plots. First, the critical exponent $\alpha_{c}=1+2/D$ reported in the previous study actually gives the transition point in the sense that the derivative of the Fisher information with respect to the exponent α is discontinuous only at $\alpha =\alpha_{c}$. Second, for $\alpha >\alpha_{c}$, each Fisher information monotonically decreases with increasing α, indicating that when the slope of the power spectrum of the covariance matrix is steeper than the critical slope, the coding performance deteriorates as the slope increases. This is intuitive, as a steeper slope implies that less neural activity is used in the coding.

However, the third and counterintuitive point is that the Fisher information is kept constant, and the coding does not degrade its performance for $\alpha <\alpha_{c}$, where the slope of the power-law decay is gentler than the critical slope. In this regime, the map from the input space to the neural space defined by the coding Eq. **1** is nondifferentiable and fractal, even in the absence of noise. This result is different from what was previously thought. The Riemann zeta function ζ(α−2/D) in Eq. **9** does indeed diverge to infinity when $\alpha \leq \alpha_{c}$ because the zeta function ζ(x) diverges to infinity for x≤1. However, this does not imply either the divergence or vanishing of the Fisher information. Rather, the information is held constant at $1/\sigma_{1}^{2}$, regardless of the neural noise strength $\sigma_{0}$. Thus, contrary to the previous conjecture, the coding performance is not directly related to the differentiability of the map defined by the coding.

Why does the coding not degrade its performance even in the fractal regime where the code is extremely sensitive to the perturbation? As shown in the previous work, the map of the coding is indeed nondifferentiable for $\alpha \leq \alpha_{c}$. However, this nondifferentiability, induced by the decrease of the power-law slope, is due to the increase of the variance of the eigenspectrum of the components with larger n, i.e. higher frequencies. Therefore, importantly, the activities of the lower frequency components relatively remain intact. Thus, by using a decoder that appropriately focuses on the directions of the low-frequency components, one can still decode enough information even from the fractal neural coding, which means that the neural activity still contains enough information of the input signals and its coding performance remains intact.

To confirm these theoretical predictions and validate the above speculation, we measured the variance of the estimation error for the input stimulus by directly constructing a maximum likelihood decoder for power-law codes. We first verified that the estimated inverse of the error variance agrees well with the analytical predictions (*SI Appendix*, section 5 and Figs. S2 and S3 for details). The inverse of the variance monotonically converges to the predicted Fisher information given by Eq. **8** in the limit of a large number of neurons.

**Fig. 4.**
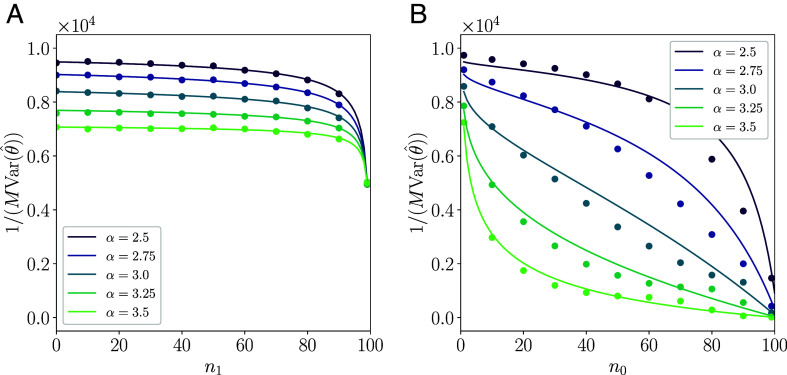
Estimation error of the maximum likelihood estimator with a limited number of encoder neurons. The vertical axis represents the inverse of the variance of the estimation error. Using direct numerical integration of the posterior distribution for D=1, we obtained the variance from $10^{5}$ realizations of the numerical estimation for various values of the exponent α (see *SI Appendix*, section 5 and Figs. S2 and S3 for details). Here, the critical exponent is $\alpha_{c}=3$, and M=10 is used. (*A*) The inverse of the variance when only encoder neurons with indices from 1 to $N-n_{1}$ are used, that is, higher-frequency mode neurons are removed first. (*B*) The same as (*A*), but only encoder neurons with indices from $n_{0}$ to N are used. That is, lower-frequency mode neurons are removed first.

We then performed the same estimation while limiting the number of available encoder neurons in the network. Fig. 4 shows the results. In these experiments, we removed neurons from the network in either descending or ascending order of their indices, n. Since neurons with larger indices encode higher frequency modes of the input, removing neurons in descending order forces the decoder to focus only on lower frequency modes (the *Left* panel of Fig. 4). In this scenario, the Fisher information remains almost unchanged compared to the case with no limitations until the final stage, where it suddenly decreases as lower-mode neurons are removed. Consequently, the estimation error increases rapidly as lower-mode neurons are removed. In contrast, when we remove neurons in ascending order of their indices (the *Right* panel of Fig. 4), the Fisher information exhibits larger decays that occur in the earlier stages of removal. These results indicate that the estimation accuracy is greatly degraded by the removal of neurons encoding low frequency components.

### Energy–Information Tradeoff Optimizes the Cortical Critical Power Law.

A natural question then is why the brain appears to operate at the critical exponent $\alpha_{c}$ rather than one of the other smaller values of α that can also achieve the best coding performance. So far, we have not considered metabolic, or energetic, costs required for the neural activities (37, 38). However, in fact, the smaller α implies that the larger amount of neural activity is evoked to represent the input signals, which may explain the optimality of the exponent $\alpha_{c}$ for the brain’s encoding.

To confirm this, we introduce a performance measure of the signal encoding including an energetic cost of neural activity. The biologically realistic description of the metabolic cost of neural activity is beyond the scope of the present work. However, a natural definition of this will be the sum of the mean square of the response activities of all neurons over the input stimuli and the noise. In the present setting, this cost converges to the zeta function in the limit of a large number of neurons N→∞:

$$\begin{array}{c} \sum_{i=1}^{2N}\frac{1}{2\pi }\int_{0}^{2\pi }{\left\langle r_{i}\left(\theta \right)\right\rangle }_{\xi_{i},\eta }^{2}d\theta =\sum_{n=1}^{N}n^{-\alpha }\to \zeta \left(\alpha \right). \end{array}$$

Therefore, the sum of the Fisher information and the energy cost gives an energy-aware performance measure of the power-law coding

[11] $$\begin{array}{c} J_{D}\left(\alpha \right)=I_{D}\left(\theta \right)-\gamma \zeta \left(\alpha \right). \end{array}$$

The regularization parameter must satisfy γ>0 because lower energy consumption is desirable.

**Fig. 5.**
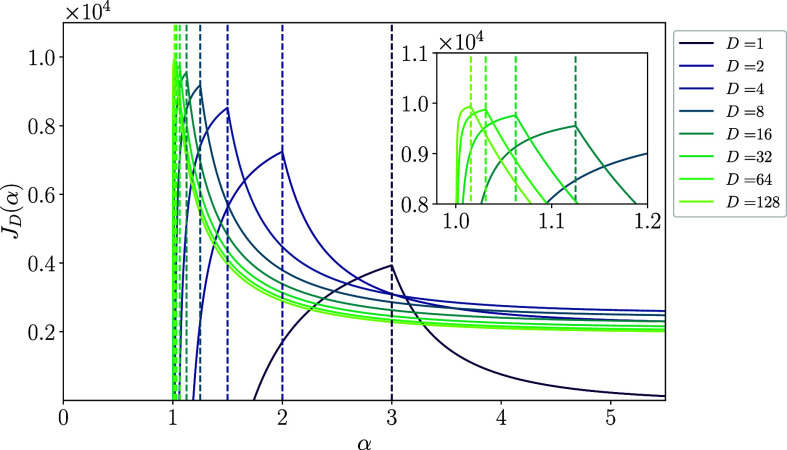
The energy-aware performance measure of the power-law coding $J_{D}$ as a function of the power-law exponent α for various values of the input dimension D. Vertical dashed lines indicate the critical values of the exponent $\alpha_{c}=1+2/D$. The *Inset* represents the magnification of the plot around α=1. Due to the energetic cost term, different from Fig. 3, regardless of input dimension, all the lines take their optimal value exactly where the power-law exponent is critical $\alpha =\alpha_{c}$. Please also note that the critical value $\alpha_{c}$ seems almost the exclusive choice of the exponent for large input dimension D as indicated by the delta-function-like curve. Here, $\sigma_{i}=\sigma_{0}=0.01$, and sufficiently small values of γ that do not overwhelm the first term of Eq. **11** are used (*SI Appendix*, section 4 for details).

Fig. 5 shows the energy-aware performance Eq. **11** as functions of the exponent α for various values of the input dimension D. In contrast to Fig. 3, the performances are maximized at the critical exponent $\alpha =\alpha_{c}$, regardless of the input dimension. This result is natural because the first term of the energy-aware performance is flat for $\alpha <\alpha_{c}$ as shown in Fig. 3, while the second term of the energy cost given by the negative zeta function is a monotonically increasing with α. In other words, while we have used arbitrarily small values of γ for the plot, the result is robust to the actual choice of values of the regularization coefficient unless it is too large to overwhelm the first term (see *SI Appendix* for details). The same argument also suggests that the energy term does not need to be the sum of the square of the neural activity to achieve the same optimality of the critical exponent, as long as the term is a monotonically increasing function of α. Thus, we can conclude that, for a broad class of energy terms, the trade-off between Fisher information and energy consumption explains the optimality of the critical exponent observed in the brain.

Instead of using the Fisher information, one may employ mutual information to evaluate the efficiency of neural coding. The mutual information between the input stimulus and the activity of encoder neurons is defined by

$$\begin{array}{c} I\left(\theta ;\;r\right)=\int d\theta \int drp\left(\theta \right)p\left(r|\theta \right)\log\frac{p(r|\theta )}{p(r)}. \end{array}$$

In the limit of a large number of encoding neurons, the mutual information can be expressed as the sum of the logarithm of the Fisher information averaged over all stimuli and the entropy of the stimulus (30):

$$\begin{array}{c} I\left(\theta ;\;r\right)=-\int d\theta p\left(\theta \right)\log p(\theta )-\int d\theta p\left(\theta \right)\frac{1}{2}\log\frac{2\pi e}{I\left(\theta \right)}. \end{array}$$

However, due to the symmetry of the receptive fields of the encoder neurons, the Fisher information is independent of the stimulus θ, as shown in Eqs. **7** and **9**. Therefore, the mutual information is a monotonically increasing function of the Fisher information. Thus, the exponent of the power-law distribution can similarly characterize the criticality and optimality of the neural code, irrespective of whether mutual information or Fisher information is used in the current framework.

## Discussion

To quantitatively evaluate the coding performance of the power-law stimulus representation in cortical neurons, we developed an analytically tractable model of neural coding. This model assumes that the spectrum of the covariance matrix follows the power law observed in the cortex (8). Using the relationship of differential correlation, which links variance and susceptibility in neural responses to input stimuli, the theory explicitly derives the Fisher information for power-law coding. Contrary to previous conjectures, our results show that neural coding performance is not degraded, even in nondifferentiable and fractal neural manifolds, where noise from input signals and neural activity strongly perturb responses. Moreover, we found that introducing a minimal metabolic cost makes the critical power-law response optimal for neural coding. Therefore, the experimentally observed power-law exponent achieves the best balance between energetic cost and Fisher information. Thus, the critical response in the brain not only balances coding expressivity and robustness but also incorporates the energetic cost, a key factor in biological computation under real-world constraints.

Our results, including the derived formula for Fisher information, essentially rely on the small-noise assumption for input perturbations and neural activities. This assumption enabled us to derive an analytical expression for the Fisher information through the Gaussian approximation of the response activity of encoding neurons, Eq. **4**. However, this assumption may not be fulfilled in the real brain. The neural activity is represented by spikes rather than firing rates, and spike responses can be quite reliable. Moreover, firing rate distributions are often better characterized by skewed distributions rather than Gaussian distributions. In the *SI Appendix*, we briefly discuss how our analytical predictions for Fisher information deviate from numerical results as noise strength increases (*SI Appendix*, section 5 and Fig. S4). When the neural noise strength $\sigma_{0}$ is increased to very large values, the discrepancy between the predictions and numerical results gradually becomes more pronounced. In contrast, the difference remains small when the input noise strength $\sigma_{1}$ increases. This is probably because the estimation variance becomes large enough to obscure the discrepancy. Extending the theory beyond the small-noise Gaussian approximation to more accurately capture the noise dependence of power-law coding remains an important direction for future research.

This study is closely related to the recent work by Bordelon and Pehlevan (39), which introduced a robust framework for analyzing the sample efficiency of learning in neural circuits. Their framework elucidated how the generalization error of linear readouts from population codes depends on factors such as the number of samples, the kernel eigenspectra of the code, and the task (39–41). Notably, they demonstrated that nondifferentiable codes could outperform differentiable ones in terms of generalization when the task is appropriately aligned with the population code and the sample size is limited. Furthermore, they showed that biological codes are metabolically more efficient than synthetic codes with equivalent structures. Their study employed a supervised framework with linear decoders to investigate the effects of sample size and generalization error. In contrast, our work uses an autoencoding approach to evaluate the intrinsic performance of the code through Fisher information, focusing primarily on the large sample size limit while considering the optimal decoder. Thus, our findings complement their work by consistently highlighting the potential of nondifferentiable biological codes from a distinct perspective. Future studies could extend our analysis to a supervised framework to uncover deeper connections to their theoretical results.

Based on the experimental observation of power-law responses in the visual cortex, this study assumes that the responses of encoding neurons can be represented by Fourier basis functions. However, neuronal responses in the brain are not restricted to Fourier-based receptive fields. For instance, Gabor filter-based receptive fields, which more accurately capture the properties of neurons in the visual cortex, could lead to modifications in the representation of Fisher information. Moreover, stimulus responses and receptive fields generally depend on sensory modalities, suggesting that the power-law exponent optimizing the tradeoff between energy and information may differ across cortical areas. Supporting this idea, recent experiments have reported slight variations in observed power-law exponents between cortical regions and species (28, 42). Therefore, extending the theory to account for stimulus representations with diverse receptive fields associated with different sensory modalities is a critical direction for future research.

Our analysis relies on experimental findings that the eigen power spectrum of the covariance matrix follows a power law with an exponent near unity. However, a recent study by Pospisil and Pillow reanalyzed the data and suggested that the earlier results may have been biased (43). Their results indicate that the eigen power spectrum is more accurately described by a broken power law with two distinct exponents. The first ten eigenvalues follow a shallow slope ($\alpha_{1}∼0.5$), while the remaining eigenvalues decay more steeply ($\alpha_{2}∼1.2$). In such a case, our expression of the Fisher information is modified by replacing its zeta function with the sum of two terms representing the two decay modes. We examined how this modification affects the behavior of the Fisher information (*SI Appendix*, section 7 and Fig. S5). The results showed that while the specific form changes, the qualitative properties, such as the information increasing with decreasing the exponents, remain intact.

In this study, we introduced the power-law dependency of neural activities a priori, assuming that each neuron independently responds to its stimulus with a power-law amplitude. This power law does not emerge as a scale-invariant feature of a multiscale system but instead reflects the response properties of individual encoding neurons to their inputs. However, the origin of this property remains unclear. Correlations in inputs or neural activities within the network may play a significant role in shaping it. Indeed, pioneering studies have shown that Fisher information is generally affected by noise correlations (32, 34, 35), while their impact tends to be small when these correlations arise from recurrent neural activities (34). Developing analyses of power-law coding by integrating theories of neural correlations in recurrent networks would be an important direction for future research (44–46). Additionally, the temporal structure of neural responses, such as transient neural activities, may also play a crucial role in these cases (47, 48).

Principal component analysis (PCA), which is based on the covariance matrix of neural activity, is limited to capturing only the linear structure of the neural manifold. However, neural manifolds may generally exhibit higher-order structures that cannot be fully represented by PCA (49). For example, geometric indices such as the curvature of the neural manifold can play an important role in determining optimal stimulus encoding. These geometric features could complement the power-law exponent of the eigenspectrum of the covariance matrix in describing the neural manifold. The Fisher information matrix provides a natural metric for statistical manifolds of probability distributions. Therefore, it may have the potential to extend the theory of optimal neural coding by incorporating higher-order structures. Exploring these aspects would be a meaningful direction for future research.

## Materials and Methods

In this section, we outline the derivation of the Fisher information (Eqs. **7** and **8**) for power-law coding. The derivation is based on the small noise assumption, which allows for a Gaussian approximation of the probability density function of the activity of encoding neurons. Additional details, including the generalization to higher input dimensions, are provided in the *SI Appendix*.

The neural activity is expressed as

$$\begin{array}{c} r=r\left(\theta +\eta \right)+\xi , \end{array}$$

where $r_{2n-1}\left(\theta \right)=n^{-\alpha /2}\cos n\theta$ and $r_{2n}\left(\theta \right)=n^{-\alpha /2}\sin n\theta$. Assuming that the neural noise strength $\sigma_{0}$ and the input noise strength $\sigma_{1}$ are sufficiently small, the activity can be linearly approximated as

$$\begin{array}{cc} r & =r\left(\theta \right)+\frac{\partial r\left(\theta \right)}{\partial \theta }\eta +\xi \\ & =m+\mu \eta +\xi . \end{array}$$

Here, m represents the mean of the neural activity, and μ is the susceptibility of the neural activity to the input signal. This expression gives the relationship between the covariance matrix and the susceptibility as

$$\begin{array}{c} \begin{array}{cc} \Sigma & ={\langle \left(r-m\right){\left(r-m\right)}^{⊤}\rangle }_{\eta ,\xi }\\ & =\sigma_{0}^{2}I+\sigma_{1}^{2}\mu \mu^{⊤}. \end{array} \end{array}$$

Furthermore, this relation leads to the interesting result that the susceptibility is an eigenvector of the covariance matrix, with its eigenvalue given by the generalized harmonic function $H_{N}\left(x\right)$, which converges to the Riemann zeta function ζ(x) as N→∞:

$$\begin{array}{c} \Sigma \mu =\left(\sigma_{0}^{2}I+\sigma_{1}^{2}\mu \mu^{⊤}\right)\mu =\left(\sigma_{0}^{2}+\sigma_{1}^{2}\mu^{⊤}\mu \right)\mu =\lambda \mu , \end{array}$$

where

[12] $$\begin{array}{c} \begin{array}{cc} \lambda & :=\sigma_{0}^{2}+\sigma_{1}^{2}\mu^{⊤}\mu \\ & =\sigma_{0}^{2}+\sigma_{1}^{2}H_{N}\left(\alpha -2\right). \end{array} \end{array}$$

From this relationship, the inverse of the covariance matrix can be explicitly expressed as

$$\begin{array}{c} \Sigma^{-1}=\frac{1}{\sigma_{0}^{2}}\left(I-\frac{\sigma_{1}^{2}}{\lambda }\mu \mu^{⊤}\right). \end{array}$$

By substituting the inverse covariance matrix into Eq. **4** and differentiating with respect to θ, we obtain the score function:

$$\begin{array}{c} \begin{array}{cc} \frac{\partial }{\partial \theta }\log p\left(r;\;\theta \right) & \approx \frac{1}{\sigma_{0}^{2}}\left({\left(r-m\right)}^{⊤}\mu -\frac{\sigma_{1}^{2}}{\lambda }{\left(r-m\right)}^{⊤}\mu \mu^{⊤}\mu \right)\\ & =\frac{1}{\sigma_{0}^{2}}\left(1-\frac{\sigma_{1}^{2}}{\lambda }\mu^{⊤}\mu \right){\left(r-m\right)}^{⊤}\mu \\ & =\frac{1}{\lambda }{\left(r-m\right)}^{⊤}\mu . \end{array} \end{array}$$

Differentiating the score function again, while using Eq. **12**, yields the Fisher information Eq. **7**:

$$\begin{array}{cc} I\left(\theta \right) & =-{\langle \frac{\partial^{2}}{\partial \theta^{2}}\log p\left(r;\;\theta \right)\rangle }_{r}\\ & =-\frac{1}{\lambda }{\langle \frac{\partial }{\partial \theta }\left({\left(r-m\right)}^{⊤}\mu \right)\rangle }_{r}\\ & =\frac{H_{N}\left(\alpha -2\right)}{\sigma_{0}^{2}+\sigma_{1}^{2}H_{N}\left(\alpha -2\right)}. \end{array}$$

In the limit of a large number of encoding neurons, this expression converges to

$$\begin{array}{c} \frac{\zeta \left(\alpha -2\right)}{\sigma_{0}^{2}+\sigma_{1}^{2}\zeta \left(\alpha -2\right)}, \end{array}$$

which is Eq. **8**

## Supplementary Material

Appendix 01 (PDF)

## Data Availability

The code used in this work is available at GitHub repository (https://github.com/tatsukawa/power-law-fisher-info) (50). All study data are included in the article and/or *SI Appendix*.
