# Supplementary material for: The cortical critical power law balances energy and information in an optimal fashion
Source: Proc Natl Acad Sci U S A. 2025 May 23;122(21):e2418218122. doi: 10.1073/pnas.2418218122 (PMC12130854; doi:10.1073/pnas.2418218122)
Supplement: Supplementary file 1 — Appendix 01 (PDF) [file pnas.2418218122.sapp.pdf]

PNAS

Supporting Information for

The cortical critical power law balances energy and information in an optimal fashion

Tsuyoshi Tatsukawa and Jun-nosuke Teramae

Jun-nosuke Teramae
E-mail: teramae@acs.i.kyoto-u.ac.jp

This PDF file includes:

Supporting text
Figs. S1 to S5
SI References

Supporting Information Text

1. Derivation of the probability density function of neural activities for the power-law coding model

In this section, we will explain how to derive the probability density function (Eq. (3) in the main text) of neural activities for the power-law coding model (Eq. (1) in the main text).

The probability density of neural activities can be obtained through the change of variables from the $2N+1$ Gaussian variables, η and ξ_i $(i = 1, \dots, 2N)$, to $2N$ neural variables, r_i $(i = 1, \dots, 2N)$. To achieve this, we introduce an auxiliary variable $\phi = \theta + \eta$ representing the noisy input to the neurons and then marginalize it. For simplicity, we denote $r_{2n-1} = x_n$ and $r_{2n} = y_n$.

$$\begin{aligned} p(\boldsymbol{r};\theta) &= p(\boldsymbol{x},\boldsymbol{y};\theta) \\ &= \int_{-\infty}^{\infty} d\phi p(\boldsymbol{x},\boldsymbol{y},\phi;\theta) \\ &= \int_{-\infty}^{\infty} d\phi p(\boldsymbol{\xi},\eta;\theta) \left| \frac{\partial(\boldsymbol{x},\boldsymbol{y},\phi)}{\partial(\boldsymbol{\xi},\eta)} \right|^{-1} \\ &= \int_{-\infty}^{\infty} d\phi p_\eta(\eta;\theta) \prod_{i=1}^{2N} p_\xi(\xi_i) \left| \frac{\partial(\boldsymbol{x},\boldsymbol{y},\phi)}{\partial(\boldsymbol{\xi},\eta)} \right|^{-1} \\ &= \int_{-\infty}^{\infty} d\phi p_\eta(\phi-\theta) \prod_{n=1}^{N} p_\xi(x_n - n^{-\alpha/2}\cos n\phi) p_\xi(y_n - n^{-\alpha/2}\sin n\phi) \\ &= \frac{1}{(2\pi\sigma_1^2)^{1/2}(2\pi\sigma_0^2)^N} \int_{-\infty}^{\infty} d\phi \exp\left[-\frac{1}{2\sigma_1^2}(\phi-\theta)^2 - \frac{1}{2\sigma_0^2}\sum_{n=1}^{N}\left(\left(x_n - n^{-\alpha/2}\cos n\phi\right)^2 + \left(y_n - n^{-\alpha/2}\sin n\phi\right)^2 \right) \right]. \end{aligned} \quad \text{[S1]}$$

Here, we have used the independence between Gaussian noise in the fourth line and the fact that the Jacobian satisfies $\left|\frac{\partial(\boldsymbol{x},\boldsymbol{y},\phi)}{\partial(\boldsymbol{\xi},\eta)}\right| = 1$ in the fifth line. This gives the desired density function, Eq. (3) in the main text.

2. Derivation of the Fisher information of the power-law coding for the case of one-dimensional input

Here, we will derive the Fisher information (Eqs. (7) and (8) in the main text) of the power-law coding via the Gaussian approximation of the probability density function of the neural activities.

A. Gaussian approximation of the probability density function. Let us assume that the neural noise strength σ_0 and the input noise strength σ_1 are sufficiently small. Then, we can expand neural activity (Eq. (1) in the main text) and approximate its probability density Eq. (S1) as a multivariate Gaussian distribution, which corresponds to the second-order approximation of the exponent of the exponential function of Eq. (S1).

The Gaussian distribution is characterized by the mean vector $\boldsymbol{m}$ and the covariance matrix Σ of the neural activities $\boldsymbol{r}$. To derive their expressions, let us denote the neural activity as

$$\boldsymbol{r} = \boldsymbol{r}(\theta + \eta) + \boldsymbol{\xi}, \quad \text{[S2]}$$

where $r_{2n-1}(\theta) = n^{-\alpha/2}\cos n\theta$ and $r_{2n}(\theta) = n^{-\alpha/2}\sin n\theta$ in the current case. Then, the linear approximation gives

$$\boldsymbol{r} = \boldsymbol{r}(\theta) + \frac{\partial \boldsymbol{r}(\theta)}{\partial\theta}\eta + \boldsymbol{\xi}. \quad \text{[S3]}$$

Therefore, by averaging this over the noise, we have the mean vector

$$\boldsymbol{m} = \langle \boldsymbol{r} \rangle_{\eta,\boldsymbol{\xi}} = \boldsymbol{r}(\theta). \quad \text{[S4]}$$

Now, let us introduce the susceptibility of the neural activity to input signal that is defined by the derivative of the mean $\boldsymbol{m}$ with respect to θ,

$$\boldsymbol{\mu} = \frac{\partial \boldsymbol{m}}{\partial\theta} = \frac{\partial \boldsymbol{r}(\theta)}{\partial\theta}. \quad \text{[S5]}$$

Using the mean and the susceptibility, we can rewrite Eq. (S3) to

$$\boldsymbol{r} = \boldsymbol{m} + \boldsymbol{\mu}\eta + \boldsymbol{\xi}. \tag{S6}$$

This expression of the neural activity allows us to have the covariance matrix using the susceptibility as

$$\begin{aligned}\Sigma &= \left\langle (\boldsymbol{r}-\boldsymbol{m})(\boldsymbol{r}-\boldsymbol{m})^\top \right\rangle_{\eta,\boldsymbol{\xi}} \\ &= \left\langle (\boldsymbol{\mu}\eta+\boldsymbol{\xi})(\boldsymbol{\mu}\eta+\boldsymbol{\xi})^\top \right\rangle_{\eta,\boldsymbol{\xi}} \\ &= \left\langle \boldsymbol{\xi}\boldsymbol{\xi}^\top \right\rangle_{\boldsymbol{\xi}} + \left\langle \eta^2 \right\rangle_\eta \boldsymbol{\mu}\boldsymbol{\mu}^\top \\ &= \sigma_0^2 \boldsymbol{I} + \sigma_1^2 \boldsymbol{\mu}\boldsymbol{\mu}^\top.\end{aligned} \tag{S7}$$

The second term of this covariance expression is called differential correlation, and it has been studied to uncover the mechanisms that constrain the information represented in neural coding.

This relation Eq. (S7) gives the interesting result that the susceptibility $\boldsymbol{\mu}$ is an eigenvector of the covariance matrix, and, in the current case, its eigenvalue is given by the generalized harmonic function $H_N(x) = \sum_{n=1}^{N} n^{-x}$ and, thus, by the Riemann zeta function $\zeta(x) = \sum_{n=1}^{\infty} n^{-x}$ in the limit of large numbers of neurons:

$$\Sigma\boldsymbol{\mu} = \left(\sigma_0^2\boldsymbol{I} + \sigma_1^2\boldsymbol{\mu}\boldsymbol{\mu}^\top\right)\boldsymbol{\mu} = \left(\sigma_0^2 + \sigma_1^2\boldsymbol{\mu}^\top\boldsymbol{\mu}\right)\boldsymbol{\mu} = \lambda\boldsymbol{\mu}, \tag{S8}$$

where

$$\begin{aligned}\lambda &:= \sigma_0^2 + \sigma_1^2\boldsymbol{\mu}^\top\boldsymbol{\mu} \\ &= \sigma_0^2 + \sigma_1^2 \sum_{n=1}^{N} n^{2-\alpha}\left(\sin^2(n\theta) + \cos^2(n\theta)\right) \\ &= \sigma_0^2 + \sigma_1^2 \sum_{n=1}^{N} n^{2-\alpha} \\ &= \sigma_0^2 + \sigma_1^2 H_N(\alpha-2) \xrightarrow[N\to\infty]{} \sigma_0^2 + \sigma_1^2\zeta(\alpha-2).\end{aligned} \tag{S9}$$

Here, we have used

$$\mu_i = \frac{\partial r_i}{\partial\theta} = \begin{cases} -n^{1-\alpha/2}\sin(n\theta) & (i = 2n-1) \\ n^{1-\alpha/2}\cos(n\theta) & (i = 2n) \end{cases}, \tag{S10}$$

because $r_{2n-1}(\theta) = n^{-\alpha/2}\cos n\theta$ and $r_{2n}(\theta) = n^{-\alpha/2}\sin n\theta$ in our case. We will see soon that the equation Eq. (S8) and Eq. (S9) will play a key role in the derivation of the Fisher information. (Note that remaining $N-1$ eigenvectors are provided by a basis spanning the $N-1$ dimensional subspace defined by $\boldsymbol{\mu}^\top\boldsymbol{x} = 0$, and their eigenvalues are degenerate with σ_0^2.)

By using the mean Eq. (S4) and the covariance Eq. (S7), the Gaussian probability density function of the neural activity is given by

$$p(\boldsymbol{r};\theta) = \frac{1}{\sqrt{(2\pi)^{2N}|\Sigma|}}\exp\left(-\frac{1}{2}(\boldsymbol{r}-\boldsymbol{m})^\top\Sigma^{-1}(\boldsymbol{r}-\boldsymbol{m})\right), \tag{S11}$$

where $|\boldsymbol{A}|$ denotes the determinant of matrix $\boldsymbol{A}$ and

$$\Sigma^{-1} = \frac{1}{\sigma_0^2}\left(I - \frac{\sigma_1^2}{\lambda}\boldsymbol{\mu}\boldsymbol{\mu}^\top\right) \tag{S12}$$

is the inverse of the covariance matrix. Eq. (S12) follows from a direct calculation using Eq. (S7) and Eq. (S8).

B. The fisher information of the power-law coding under the Gaussian approximation. The density function Eq. (S11) allows us to have the loglikelihood function of θ given the neural activities $\boldsymbol{r}$,

$$\begin{aligned}\log p(\boldsymbol{r};\theta) &= -\frac{1}{2}(\boldsymbol{r}-\boldsymbol{m})^\top \Sigma^{-1}(\boldsymbol{r}-\boldsymbol{m}) + C \\ &= -\frac{1}{2\sigma_0^2}(\boldsymbol{r}-\boldsymbol{m})^\top \left(I - \frac{\sigma_1^2}{\lambda}\boldsymbol{\mu}\boldsymbol{\mu}^\top\right)(\boldsymbol{r}-\boldsymbol{m}) + C \\ &= -\frac{1}{2\sigma_0^2}\left(|\boldsymbol{r}-\boldsymbol{m}|^2 - \frac{\sigma_1^2}{\lambda}\left(\boldsymbol{\mu}^\top(\boldsymbol{r}-\boldsymbol{m})\right)^2\right) + C,\end{aligned} \tag{S13}$$

where C denotes the term not including the input stimulus θ. Thus, the score function, i.e., the derivative of the loglikelihood with respect to the input θ is given by

$$\begin{aligned}\frac{\partial}{\partial\theta}\log p(\boldsymbol{r};\theta) &= -\frac{1}{2\sigma_0^2}\left(\frac{\partial}{\partial\theta}|\boldsymbol{r}-\boldsymbol{m}|^2 - \frac{\sigma_1^2}{\lambda}\frac{\partial}{\partial\theta}\left((\boldsymbol{r}-\boldsymbol{m})^\top\boldsymbol{\mu}\right)^2\right) \\ &= -\frac{1}{\sigma_0^2}\left(-(\boldsymbol{r}-\boldsymbol{m})^\top\frac{\partial\boldsymbol{m}}{\partial\theta} - \frac{\sigma_1^2}{\lambda}(\boldsymbol{r}-\boldsymbol{m})^\top\boldsymbol{\mu}\left(-\frac{\partial\boldsymbol{m}}{\partial\theta}^\top\boldsymbol{\mu} + (\boldsymbol{r}-\boldsymbol{m})^\top\frac{\partial\boldsymbol{\mu}}{\partial\theta}\right)\right) \\ &\approx \frac{1}{\sigma_0^2}\left((\boldsymbol{r}-\boldsymbol{m})^\top\boldsymbol{\mu} - \frac{\sigma_1^2}{\lambda}(\boldsymbol{r}-\boldsymbol{m})^\top\boldsymbol{\mu}\boldsymbol{\mu}^\top\boldsymbol{\mu}\right) \\ &= \frac{1}{\sigma_0^2}\left(1 - \frac{\sigma_1^2}{\lambda}\boldsymbol{\mu}^\top\boldsymbol{\mu}\right)(\boldsymbol{r}-\boldsymbol{m})^\top\boldsymbol{\mu} \\ &= \frac{1}{\lambda}(\boldsymbol{r}-\boldsymbol{m})^\top\boldsymbol{\mu}.\end{aligned} \tag{S14}$$

To obtain the third line, we used Eq. (S5) and omitted the higher-order term of $\boldsymbol{r}-\boldsymbol{m}$, as it only contributes a higher-order correction to the Fisher information, which vanishes under the small noise approximation*. The last line follows from the first line of Eq. (S9).

By differentiating the score function again, we arrive at the Fisher information of the power-law coding

$$\begin{aligned}I(\theta) &= -\left\langle \frac{\partial^2}{\partial\theta^2}\log p(\boldsymbol{r};\theta)\right\rangle_{\boldsymbol{r}} \\ &= -\frac{1}{\lambda}\left\langle\frac{\partial}{\partial\theta}\left((\boldsymbol{r}-\boldsymbol{m})^\top\boldsymbol{\mu}\right)\right\rangle_{\boldsymbol{r}} \\ &= \frac{1}{\lambda}\boldsymbol{\mu}^\top\boldsymbol{\mu} \\ &= \frac{|\boldsymbol{\mu}|^2}{\sigma_0^2 + \sigma_1^2|\boldsymbol{\mu}|^2} \\ &= \frac{H_N(\alpha-2)}{\sigma_0^2 + \sigma_1^2 H_N(\alpha-2)}.\end{aligned} \tag{S15}$$

The last line follows from Eq. (S9). In the limit of a large number of N, the fisher information converges to

$$\frac{\zeta(\alpha-2)}{\sigma_0^2 + \sigma_1^2\zeta(\alpha-2)}, \tag{S16}$$

which is Eq. (8) in the main text. We used Eq. (S15) to plot the dotted lines of Fig. S3 and used Eq. (S16) for the thick line.

*The term we have omitted is

$$\frac{1}{\lambda}\frac{\sigma_1^2}{\sigma_0^2}\left(\boldsymbol{\mu}^\top(\boldsymbol{r}-\boldsymbol{m})(\boldsymbol{r}-\boldsymbol{m})^\top\frac{\partial\boldsymbol{\mu}}{\partial\theta}\right),$$

whose differentiation with respect to θ yields

$$\frac{1}{\lambda}\frac{\sigma_1^2}{\sigma_0^2}\left(\frac{\partial\boldsymbol{\mu}}{\partial\theta}^\top(\boldsymbol{r}-\boldsymbol{m})(\boldsymbol{r}-\boldsymbol{m})^\top\frac{\partial\boldsymbol{\mu}}{\partial\theta} - \boldsymbol{\mu}^\top\boldsymbol{\mu}(\boldsymbol{r}-\boldsymbol{m})^\top\frac{\partial\boldsymbol{\mu}}{\partial\theta} - \boldsymbol{\mu}^\top(\boldsymbol{r}-\boldsymbol{m})\boldsymbol{\mu}^\top\frac{\partial\boldsymbol{\mu}}{\partial\theta} + \boldsymbol{\mu}^\top(\boldsymbol{r}-\boldsymbol{m})(\boldsymbol{r}-\boldsymbol{m})^\top\frac{\partial^2\boldsymbol{\mu}}{\partial\theta^2}\right).$$

By averaging this term using Eq. (S4) and Eq. (S7), we find that this omitted term contributes an additional term

$$\frac{1}{\lambda}\frac{\sigma_1^2}{\sigma_0^2}\left(\frac{\partial\boldsymbol{\mu}}{\partial\theta}^\top\Sigma\frac{\partial\boldsymbol{\mu}}{\partial\theta} - 0 - 0 + \boldsymbol{\mu}^\top\Sigma\frac{\partial^2\boldsymbol{\mu}}{\partial\theta^2}\right) = \frac{1}{\lambda}\frac{\sigma_1^2}{\sigma_0^2}\left(\sigma_0^2\left(\left|\frac{\partial\boldsymbol{\mu}}{\partial\theta}\right|^2 + \boldsymbol{\mu}^\top\frac{\partial^2\boldsymbol{\mu}}{\partial\theta^2}\right) + \sigma_1^2\left(\left|\boldsymbol{\mu}^\top\frac{\partial\boldsymbol{\mu}}{\partial\theta}\right|^2 + \boldsymbol{\mu}^\top\boldsymbol{\mu}\boldsymbol{\mu}^\top\frac{\partial^2\boldsymbol{\mu}}{\partial\theta^2}\right)\right)$$

to the Fisher information Eq. (S15). However, this contribution is negligibly small compared to the dominant term of the Fisher information shown in Eq. (S15) under the small noise assumption we have employed ($\sigma_0, \sigma_1 \ll 1$).

3. Derivation of the Fisher information of the power-law coding for high dimensional input stimulus

In this section, extending the results of the above sections, we derive the Fisher information of the power-law coding for a multidimensional input stimulus.

To simplify the calculation, instead of using the trigonometric functions $\cos n\theta$ and $\sin n\theta$ $(n = 1, \cdots, N)$ to represent neural activity, let us introduce complex Fourier basis functions $e^{in\theta}/\sqrt{2}$ $(n = \pm 1, \ldots, \pm N)$ that satisfies the constraint $z_{-n} = \overline{z_n}$, where $\overline{z}$ is the complex conjugate of z. The factor $1/\sqrt{2}$ appears here to make the norm of the complex basis functions equal to that of the trigonometric functions in function space because it holds that

$$\int_0^{2\pi} |\cos n\theta|^2 \, d\theta = \int_0^{2\pi} |\sin n\theta|^2 \, d\theta = \int_0^{2\pi} \left| \frac{1}{\sqrt{2}} e^{in\theta} \right|^2 d\theta.$$

The neural activities for one dimensional input is thus rewritten by

$$r_n = z_n = \frac{1}{2^{1/2}} |n|^{-\alpha/2} e^{in(\theta+\eta)} + \xi_n, \tag{S17}$$

where ξ_n is the complex Gaussian variable with the mean 0 and the strength σ_0, namely, its real and imaginary parts independently follow the Gaussian distribution of the mean 0 and the variance $\sigma_0^2/2$ with satisfying $\xi_{-n} = \overline{\xi_n}$, which gives $\langle \xi_n \xi_{-m} \rangle = \delta_{nm} \langle \xi_n \overline{\xi_n} \rangle = \delta_{nm} \langle (\mathrm{Re}\, \xi_n)^2 + (\mathrm{Im}\, \xi_n)^2 \rangle = \sigma_0^2 \delta_{nm}$.

Similarly, by regarding the neural representations of the D-dimensional input $\boldsymbol{\theta} = (\theta_1, \ldots \theta_D)^\top$ as the multidimensional (complex) Fourier expansion, we can extend the above neural activity to

$$z_{\boldsymbol{k}} = \frac{1}{2^{D/2}} n(\boldsymbol{k})^{-\alpha/2} e^{i\boldsymbol{k}^\top(\boldsymbol{\theta}+\boldsymbol{\eta})} + \xi_{\boldsymbol{k}}, \tag{S18}$$

where each neuron is indexed by D-dimensional lattice vectors $\boldsymbol{k} = (k_1, \ldots, k_D)^\top$, where k_d $(d = 1, \ldots, D)$ is the integer representing the frequency or the wavenumber of the neural activity for the dth input stimulus θ_d. The function $n(\cdot)$ is a numbering that aligns the neurons in ascending order of their frequencies, and thus a function of the lattice vector $\boldsymbol{k}$. For now, we leave it as an arbitrary function satisfying the condition

$$n(k_1, \ldots, -k_d, \ldots, k_D) = n(k_1, \ldots, k_d, \ldots, k_D) \tag{S19}$$

for $d = 1, \ldots, D$, and will specify it later. Input noise $\boldsymbol{\eta} = (\eta_1, \ldots \eta_D)^\top$ and neural noise $\xi_{\boldsymbol{k}}$ are real and complex independent Gaussian variables, respectively, satisfying $\langle \eta_d \rangle = \langle \xi_{\boldsymbol{k}} \rangle = \langle \eta_d \xi_{\boldsymbol{k}} \rangle = 0$, $\langle \eta_d \eta_k \rangle = \sigma_d^2 \delta_{dk}$, and $\langle (\mathrm{Re}\, \xi_{\boldsymbol{k}})^2 \rangle = \langle (\mathrm{Im}\, \xi_{\boldsymbol{k}})^2 \rangle = \sigma_0^2/2$, and thus,

$$\langle \xi_{\boldsymbol{k}} \xi_{-\boldsymbol{l}} \rangle = \delta_{\boldsymbol{kl}} \left\langle \xi_{\boldsymbol{k}} \overline{\xi_{\boldsymbol{k}}} \right\rangle = \delta_{\boldsymbol{kl}} \left\langle (\mathrm{Re}\, \xi_{\boldsymbol{k}})^2 + (\mathrm{Im}\, \xi_{\boldsymbol{k}})^2 \right\rangle = \sigma_0^2 \delta_{\boldsymbol{kl}},$$

where $\delta_{\boldsymbol{kl}}$ means $\delta_{k_1 l_1} \cdots \delta_{k_D l_D}$, and σ_0 and σ_d are the strengths of the neural and dth input, respectively. The first equality of the above follows from $\xi_{-\boldsymbol{k}} = \overline{\xi_{\boldsymbol{k}}}$ that is required for $z_{-\boldsymbol{k}} = \overline{z_{\boldsymbol{k}}}$.

Similar to the previous sections, by assuming that the noise strengths are sufficiently small, we approximate the density function of the neural activities with the multivariate (complex) Gaussian distribution

$$p(\boldsymbol{z}; \theta) \propto \exp\left(-\frac{1}{2} (\boldsymbol{z} - \boldsymbol{m})^* \Sigma^{-1} (\boldsymbol{z} - \boldsymbol{m}) \right). \tag{S20}$$

Here, we defined a column vector $\boldsymbol{z}$ by reshaping $z_{\boldsymbol{k}} = z_{k_1 \ldots k_D}$ that is indeed a tensor as indicated by the multiple subscripts. Thus, for instance,

$$\boldsymbol{z} = \begin{pmatrix} z_{-K_1, \ldots, -K_D} \\ z_{-K_1, \ldots, -K_D+1} \\ \vdots \\ z_{K_1, \ldots, K_D} \end{pmatrix}. \tag{S21}$$

The complex mean vector $\boldsymbol{m}$ and the complex covariance matrix Σ of $\boldsymbol{z}$ are defined by

$$\boldsymbol{m} = \langle \boldsymbol{z} \rangle \tag{S22}$$

$$\Sigma = \langle (\boldsymbol{z} - \boldsymbol{m})(\boldsymbol{z} - \boldsymbol{m})^* \rangle, \tag{S23}$$

where $\boldsymbol{x}^*$ denotes the Hermitian conjugate of the vector $\boldsymbol{x}$. While $\boldsymbol{z}$ is a column vector as defined above, we continue to abuse notation of the tensor index $z_{\boldsymbol{k}} = z_{k_1 \dots k_D}$ to denote its component as before for convenience. Similarly, we use $m_{\boldsymbol{k}} = m_{k_1 \dots k_D}$ and $\Sigma_{\boldsymbol{kl}} = \Sigma_{k_1 \dots k_D, l_1 \dots l_D}$ to denote components of the vector $\boldsymbol{m}$ and the matrix Σ, respectively.

The same argument as before gives the linear approximation of the complex neural activity as

$$\boldsymbol{z} = \boldsymbol{m} + \sum_{d=1}^{D} \boldsymbol{\mu}_d \eta_d + \boldsymbol{\xi}, \tag{S24}$$

where components of the complex mean $\boldsymbol{m} = (m_{\boldsymbol{k}})$ is given by

$$m_{\boldsymbol{k}} = \langle z_{\boldsymbol{k}} \rangle = \frac{1}{2^{D/2}} n(\boldsymbol{k})^{-\alpha/2} e^{i\boldsymbol{k}^\top \boldsymbol{\theta}} \tag{S25}$$

and the susceptibility $\boldsymbol{\mu}_d$ $(d = 1, \dots, D)$ is defined by the derivative the vector $\boldsymbol{m}$ with respect to the dth input signal θ_d

$$\boldsymbol{\mu}_d := \frac{\partial \boldsymbol{m}}{\partial \theta_d} \tag{S26}$$

whose components are given by

$$(\boldsymbol{\mu}_d)_{\boldsymbol{k}} = \frac{\partial m_{\boldsymbol{k}}}{\partial \theta_d} = ik_d m_{\boldsymbol{k}} = ik_d \frac{1}{2^{D/2}} n(\boldsymbol{k})^{-\alpha/2} e^{i\boldsymbol{k}^\top \boldsymbol{\theta}}. \tag{S27}$$

Then, using the susceptibility, we obtain an expression of the covariance as

$$\begin{aligned} \Sigma &= \langle (\boldsymbol{z} - \boldsymbol{m})(\boldsymbol{z} - \boldsymbol{m})^* \rangle_{\boldsymbol{\eta},\boldsymbol{\xi}} \\ &= \left\langle \left(\sum_{d=1}^{D} \boldsymbol{\mu}_d \eta_d + \boldsymbol{\xi} \right) \left(\sum_{d=1}^{D} \boldsymbol{\mu}_d \eta_d + \boldsymbol{\xi} \right)^* \right\rangle_{\boldsymbol{\eta},\boldsymbol{\xi}} \\ &= \langle \boldsymbol{\xi}\boldsymbol{\xi}^* \rangle_{\boldsymbol{\xi}} + \sum_{d=1}^{D} \left\langle \eta_d^2 \right\rangle_{\eta_d} \boldsymbol{\mu}\boldsymbol{\mu}^* \\ &= \sigma_0^2 I + \sum_{d=1}^{D} \sigma_d^2 \boldsymbol{\mu}_d \boldsymbol{\mu}_d^*, \end{aligned} \tag{S28}$$

which associates the fluctuation and the susceptibilities of the neural activity.

Because of the condition Eq. (S19), the susceptibilities satisfy the orthogonal condition

$$\begin{aligned} \boldsymbol{\mu}_d^* \boldsymbol{\mu}_l &= \frac{1}{2^D} \sum_{\boldsymbol{k} \in \{n(\boldsymbol{k}) \le N\}} k_d k_l n(\boldsymbol{k})^{-\alpha} \\ &= \delta_{dl} \frac{1}{2^D} \sum_{\boldsymbol{k} \in \{n(\boldsymbol{k}) \le N\}} k_d^2 n(\boldsymbol{k})^{-\alpha} \\ &= \delta_{dl} |\boldsymbol{\mu}_d|^2. \end{aligned} \tag{S29}$$

Thus, we can show that the each susceptibility is an eigenvector of the complex covariance matrix

$$\Sigma \boldsymbol{\mu}_d = \left(\sigma_0^2 I + \sum_{l=1}^{D} \sigma_l^2 \boldsymbol{\mu}_l \boldsymbol{\mu}_l^* \right) \boldsymbol{\mu}_d = \left(\sigma_0^2 + \sigma_d^2 |\boldsymbol{\mu}_d|^2 \right) \boldsymbol{\mu}_d = \lambda_d \boldsymbol{\mu}_d, \tag{S30}$$

where we defined the dth eigenvalue by

$$\lambda_d := \sigma_0^2 + \sigma_d^2 |\boldsymbol{\mu}_d|^2, \tag{S31}$$

with

$$|\boldsymbol{\mu}_d|^2 = \frac{1}{2^D} \sum_{\boldsymbol{k} \in \{n(\boldsymbol{k}) \le N\}} k_d^2 n(\boldsymbol{k})^{-\alpha}. \tag{S32}$$

Direct calculation using Eq. (S30) gives the inverse of the covariance matrix as

$$\Sigma^{-1} = \frac{1}{\sigma_0^2}\left(I - \sum_{d=1}^{D} \frac{\sigma_d^2}{\lambda_d} \boldsymbol{\mu}_d \boldsymbol{\mu}_d^*\right), \tag{S33}$$

and thus, the loglikelihood function of the multidimensional input $\boldsymbol{\theta}$ given the neural activities $\boldsymbol{z}$ is given by

$$\begin{aligned}\log p(\boldsymbol{z};\boldsymbol{\theta}) &= -\frac{1}{2}(\boldsymbol{z}-\boldsymbol{m})^*\Sigma^{-1}(\boldsymbol{z}-\boldsymbol{m}) + C \\ &= -\frac{1}{2\sigma_0^2}(\boldsymbol{z}-\boldsymbol{m})^*\left(I - \sum_{d=1}^{D} \frac{\sigma_d^2}{\lambda_d} \boldsymbol{\mu}_d \boldsymbol{\mu}_d^*\right)(\boldsymbol{z}-\boldsymbol{m}) + C \\ &= -\frac{1}{2\sigma_0^2}\left(|\boldsymbol{z}-\boldsymbol{m}|^2 - \sum_{d=1}^{D} \frac{\sigma_d^2}{\lambda_d}|(\boldsymbol{z}-\boldsymbol{m})^*\boldsymbol{\mu}_d|^2\right) + C.\end{aligned} \tag{S34}$$

where C denotes the term not including $\boldsymbol{\theta}$. The derivative of the loglikelihood with respect to a component of the input vector gives the score function

$$\begin{aligned}\frac{\partial}{\partial\theta_i}\log p(\boldsymbol{z};\boldsymbol{\theta}) &= -\frac{1}{2\sigma_0^2}\left(-\frac{\partial \boldsymbol{m}^*}{\partial\theta_i}(\boldsymbol{z}-\boldsymbol{m}) - \sum_{d=1}^{D}\frac{\sigma_d^2}{\lambda_d}\left(-\frac{\partial \boldsymbol{m}^*}{\partial\theta_i}\boldsymbol{\mu}_d + (\boldsymbol{z}-\boldsymbol{m})^*\frac{\partial \boldsymbol{\mu}_d}{\partial\theta_i}\right)\boldsymbol{\mu}_d^*(\boldsymbol{z}-\boldsymbol{m}) + \text{h.c.}\right) \\ &\approx \frac{1}{2\sigma_0^2}\left(\boldsymbol{\mu}_i^*(\boldsymbol{z}-\boldsymbol{m}) - \sum_{d=1}^{D}\frac{\sigma_d^2}{\lambda_d}\boldsymbol{\mu}_i^*\boldsymbol{\mu}_d\boldsymbol{\mu}_d^*(\boldsymbol{z}-\boldsymbol{m}) + \text{h.c.}\right) \\ &= \frac{1}{2\sigma_0^2}\left(1 - \frac{\sigma_i^2|\boldsymbol{\mu}_i|^2}{\lambda_i}\right)\boldsymbol{\mu}_i^*(\boldsymbol{z}-\boldsymbol{m}) + \text{h.c.} \\ &= \frac{1}{2\lambda_i}\boldsymbol{\mu}_i^*(\boldsymbol{z}-\boldsymbol{m}) + \text{h.c.},\end{aligned} \tag{S35}$$

where h.c. denotes the Hermitian conjugate of previous terms. To obtain the second line, we used Eq. (S26) and omitted the second-order term of $\boldsymbol{z}-\boldsymbol{m}$ that will only give higher-order terms to the Fisher information which vanish anyway under the small noise assumption. The third line is from the orthogonality Eq. (S29) and the last line follows from the definition of the eigenvalue Eq. (S31).

Then, we obtain the component of the Fisher information by averaging the negative derivative of the score function

$$\begin{aligned}I_{ij}(\boldsymbol{\theta}) &= \left\langle -\frac{\partial^2}{\partial\theta_i\partial\theta_j}\log p(\boldsymbol{z};\boldsymbol{\theta})\right\rangle_{\boldsymbol{z}} \\ &= \left\langle -\frac{\partial}{\partial\theta_j}\left(\frac{1}{2\lambda_i}\boldsymbol{\mu}_i^*(\boldsymbol{z}-\boldsymbol{m}) + \text{h.c.}\right)\right\rangle_{\boldsymbol{z}} \\ &= \frac{1}{\lambda_i}\boldsymbol{\mu}_i^*\boldsymbol{\mu}_j \\ &= \frac{|\boldsymbol{\mu}_i|^2}{\lambda_i}\delta_{ij} \\ &= \frac{|\boldsymbol{\mu}_i|^2}{\sigma_0^2 + \sigma_i^2|\boldsymbol{\mu}_i|^2}\delta_{ij}.\end{aligned} \tag{S36}$$

Note that Eq. (S36) with Eq. (S32) recovers the Fisher information Eq. (S15) for the case one-dimensional input as a special case,

$$I_{11}(\theta) = \frac{|\boldsymbol{\mu}_1|^2}{\sigma_0^2 + \sigma_1^2|\boldsymbol{\mu}_1|^2} = \frac{\frac{1}{2}\sum_{k=-N}^{N}k^2k^{-\alpha}}{\sigma_0^2 + \sigma_1^2\frac{1}{2}\sum_{k=-N}^{N}k^2k^{-\alpha}} = \frac{\sum_{k=1}^{N}k^2k^{-\alpha}}{\sigma_0^2 + \sigma_1^2\sum_{k=1}^{N}k^2k^{-\alpha}} = \frac{H_N(\alpha-2)}{\sigma_0^2 + \sigma_1^2 H_N(\alpha-2)}. \tag{S37}$$

To proceed further, we need to specify the function $n(\boldsymbol{k})$ that ranks the multidimensional lattice vectors $\boldsymbol{k} = (k_1, \ldots, k_D)$ in ascending order of $\boldsymbol{k}$. Since "ascending order" is ambiguous in the multidimensional lattice space, we define the rank as it is consistent with the distance from the origin $|\boldsymbol{k}| = \sqrt{k_1^2 + \cdots + k_D^2}$. For example, we can define the function by

$$n(\boldsymbol{k}) = \sum_{\boldsymbol{h}\geq 0} \mathbb{1}_{|\boldsymbol{h}|<|\boldsymbol{k}|}[\boldsymbol{h}] = \frac{1}{2^D}\sum_{\boldsymbol{h}} \mathbb{1}_{|\boldsymbol{h}|<|\boldsymbol{k}|}[\boldsymbol{h}], \quad \text{[S38]}$$

where $\boldsymbol{h} \geq 0$ means $h_d \geq 0$ for all d, and $\mathbb{1}_A[\boldsymbol{h}]$ is the indicator function that returns one for the lattice point $\boldsymbol{h}$ satisfying the condition A and zero otherwise. Thus, the definition above means that n of the lattice point $\boldsymbol{k}$ is defined as the number of lattices $\boldsymbol{h}(\geq 0)$ whose distance from the origin is smaller than that of $\boldsymbol{k}$. In other words, $n(\boldsymbol{k})$ is equal to the number of lattice points inside the open ball with radius $|\boldsymbol{k}|$ and in the non-negative orthant. Figure S1 illustrates this for the case of $D = 2$. Note that the value of $n(\boldsymbol{k})$ for $\boldsymbol{k} \ngeq 0$ is determined by the symmetry condition Eq. (S19).

Because the sum of the right hand side of Eq. (S38) is approximated by the volume of the D-ball of radius r for large n, we have

$$n(\boldsymbol{k}) = \frac{1}{2^D} V_D\, |\boldsymbol{k}|^D\,, \quad \text{[S39]}$$

where $V_D = \pi^{D/2}/\Gamma(D/2+1)$ is the volume of the unit D-ball. It gives

$$k_1^2 + \cdots + k_D^2 = |\boldsymbol{k}|^2 = 4\Big(\frac{1}{V_D} n(\boldsymbol{k})\Big)^{2/D}. \quad \text{[S40]}$$

Then using the symmetry of the function $n(\boldsymbol{k})$, we have

$$\begin{aligned}
|\boldsymbol{\mu}_1|^2 = \cdots = |\boldsymbol{\mu}_D|^2 &= \frac{1}{D}\big(|\boldsymbol{\mu}_1|^2 + \cdots + |\boldsymbol{\mu}_D|^2\big) \\
&= \frac{1}{D2^D} \sum_{\boldsymbol{k}\in\{n(\boldsymbol{k})\leq N\}} \big(k_1^2 + \cdots + k_D^2\big) n(\boldsymbol{k})^{-\alpha} \\
&= \frac{1}{D2^D} \sum_{\boldsymbol{k}\in\{n(\boldsymbol{k})\leq N\}} |\boldsymbol{k}|^2\, n(\boldsymbol{k})^{-\alpha} \\
&= \frac{1}{D} \sum_{\boldsymbol{k}\in\{\boldsymbol{k}\geq 0 \wedge n(\boldsymbol{k})\leq N\}} |\boldsymbol{k}|^2\, n(\boldsymbol{k})^{-\alpha} \\
&= \frac{4}{D} V_D^{-2/D} \sum_{\boldsymbol{k}\in\{\boldsymbol{k}\geq 0 \wedge n(\boldsymbol{k})\leq N\}} n(\boldsymbol{k})^{-\alpha+2/D} \\
&= \frac{4}{D} V_D^{-2/D} \sum_{n=1}^{N} n^{-\alpha+2/D} \\
&= \frac{4}{D} V_D^{-2/D} H_N(\alpha - 2/D).
\end{aligned} \quad \text{[S41]}$$

By putting this to Eq. (S36), we arrive at

$$I_{ij}(\boldsymbol{\theta}) = \frac{H_N(\alpha - 2/D)}{\sigma_0^2 D V_D^{2/D}/4 + \sigma_i^2 H_N(\alpha - 2/D)} \delta_{ij}, \quad \text{[S42]}$$

which converges to

$$\frac{\zeta(\alpha - 2/D)}{\sigma_0^2 D V_D^{2/D}/4 + \sigma_i^2 \zeta(\alpha - 2/D)}, \quad \text{[S43]}$$

in the limit of $N \to \infty$, which further converges to

$$\frac{\zeta(\alpha)}{e\pi\sigma_0^2/2 + \sigma_i^2\zeta(\alpha)}, \quad \text{[S44]}$$

in the limit of $D \to \infty$ because it holds that

$$\frac{D V_D^{2/D}}{4} = \frac{D}{4}\left(\frac{\pi^{D/2}}{\Gamma(D/2+1)}\right)^{2/D} \approx \frac{D}{4}\left(\frac{\pi^{D/2}}{(\pi D)^{1/2}\left(\frac{D}{2e}\right)^{D/2}}\right)^{2/D} = \frac{e\pi}{2(\pi D)^{1/D}} \xrightarrow[D\to\infty]{} \frac{e\pi}{2} \quad \text{[S45]}$$

due to the Stirling's formula. These expressions of the Fisher information Eq. (S43) and Eq. (S44) are Eqs. (9) and (10) in the main text, respectively.

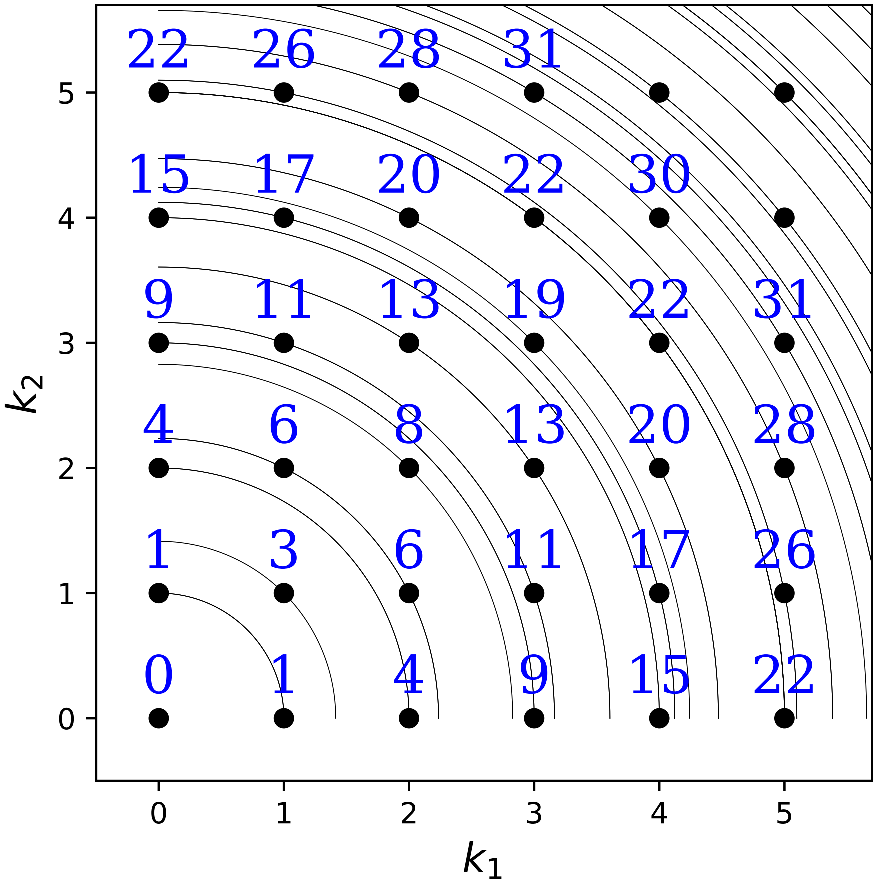

Fig. S1. An example of the numbering $n(\boldsymbol{k})$ that aligns the neurons in ascending order of their frequency $\boldsymbol{k}$ for the case of $D = 2$. The numbering function, or the rank precisely, $n(\boldsymbol{k})$ of the neuron at the lattice $\boldsymbol{k}$ is given as the numbers of neurons, or the lattices, whose distance from the origin is smaller than that of the lattice $\boldsymbol{k}$ (blue numbers). Therefore, the function $n(\boldsymbol{k})$ is given by the number of lattices inside the circle with radius $|\boldsymbol{k}|$ (black lines), whose leading order term is equal to the area of the disk enclosed by the circle, i.e., 2-ball, for large $n(\boldsymbol{k})$.

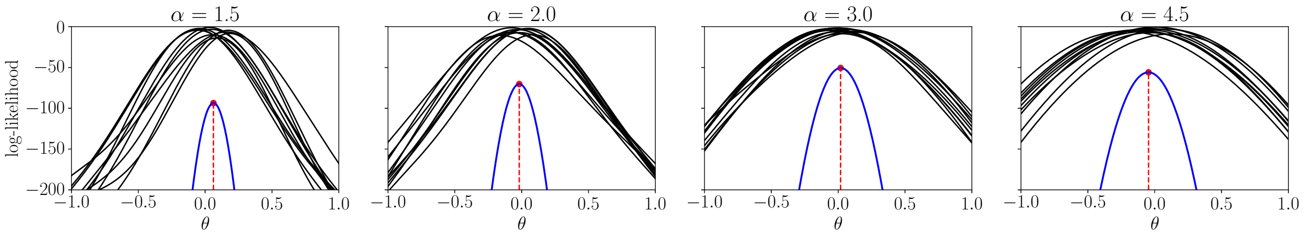

Fig. S2. Outline of the maximum likelihood estimation of the input stimulus from the power-law population coding. The logarithm of the posterior functions $\log P(r_i|\theta)$ (black lines) are directly calculated by numerical integration of the posterior distribution Eq. (S1) for various realizations of neural activities Eq. (S2) as functions of the variable θ. Each panel exemplifies the functions for values of exponent α, where we show just ten realizations of the posterior functions to illustrate the procedure. The maximum likelihood estimate $\hat{\theta}$ (the red point and the red dashed line) of the input signal is given as the value of θ that maximizes the log-likelihood function Eq. (S51) (the blue line) that is the sum of the logarithm of the posterior functions.

4. Condition for the regularization parameter of the energy-aware performance measure

For completeness, in this section we provide the condition that the energy cost given by the second term of the energy-aware performance measure $J_D(\alpha)$ (Eq. (11) in the main text) does not overwhelm the first term.

The energy-aware performance measure was given by

$$J_D(\alpha) = I_D(\alpha) - \gamma\zeta(\alpha) = \frac{\zeta(\alpha - 2/D)}{\sigma_0^2 D V_D^{2/D}/4 + \sigma_i^2 \zeta(\alpha - 2/D)} - \gamma\zeta(\alpha). \quad \text{[S46]}$$

Considering that the second term $-\gamma\zeta(\alpha)$ is a monotonically increasing function of α whereas the first term of the Fisher information is constant until it monotonically decreases from the critical point $\alpha = \alpha_c$, it is obvious that the $J_D(\alpha)$ is monotonically increasing for $\alpha < \alpha_c = 1 + 2/D$. Therefore, the condition that α_c gives the maximum of J_D is given by the one that the derivative of $J_D(\alpha)$ at $\alpha \to \alpha_c + 0$ is negative:

$$\lim_{\alpha \to \alpha_c + 0} J_D'(\alpha) = I_D'(\alpha_c + 0) - \gamma\zeta'(\alpha_c) \quad \text{[S47]}$$

$$= \frac{\sigma_0^2 D V_D^{2/D}/4\zeta'(1)}{\left(\sigma_0^2 D V_D^{2/D}/4 + \sigma_i^2 \zeta(1)\right)^2} - \gamma\zeta'(\alpha_c) < 0. \quad \text{[S48]}$$

By Solving this inequality, we obtain the condition for the regularization parameter γ as

$$\begin{aligned}\gamma &< \frac{\sigma_0^2 D V_D^{2/D}/4\zeta'(1)}{\left(\sigma_0^2 D V_D^{2/D}/4 + \sigma_i^2 \zeta(1)\right)^2 \zeta'(\alpha_c)} \\ &= -\frac{\sigma_0^2 D V_D^{2/D}}{4\sigma_i^4 \zeta'(\alpha_c)} =: \gamma_c,\end{aligned} \quad \text{[S49]}$$

where we used $\zeta'(\alpha_c) < 0$ and the formula $\zeta'(1)/(a + \zeta(1))^2 = -1$ that follows from the fact that the Riemann zeta function $\zeta(z)$ has only a simple pole at $z = 1$ with residual 1, i.e., the principal part of $\zeta(z)$ is $1/(1-z)$. Conversely, when γ satisfies the above condition, we can easily check that $J_D'(\alpha) < 0$ for all $\alpha > \alpha_c$ by using the fact that $I_D'(\alpha)$ is an increasing function there,

$$J_D'(\alpha) = I_D'(\alpha) - \gamma\zeta'(\alpha) < I_D'(\alpha) - I_D'(\alpha_c) < 0. \quad \text{[S50]}$$

We used $\gamma = \gamma_c/10$ for Fig. 4 in the main text.

5. Maximum likelihood estimator for the power-law code

To validate the theoretical predictions, we actually construct the maximum likelihood decoder for the power-law coding and directly measure the variance of the estimation errors of the input stimulus θ to compare it with the inverse of the predicted Fisher information. For a given set of M observations of neural activities $\boldsymbol{r}^{(m)} (m = 1, \ldots, M)$ responding to the input stimulus θ, the log-likelihood function of the input stimulus is given by

$$L\left(\theta; \boldsymbol{r}^{(1)}, \ldots, \boldsymbol{r}^{(M)}\right) = \sum_{m=1}^{M} \log p(\boldsymbol{r}^{(m)}; \theta), \quad \text{[S51]}$$

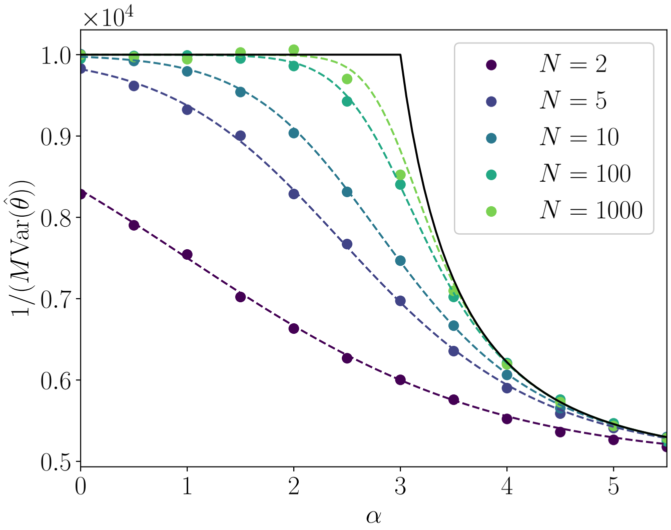

Fig. S3. The inverse of the variance of the estimation error of the maximum likelihood estimation of the input stimulus for the power-law population coding. This inverse will be the asymptotical equivalence of the Fisher information of the coding. Using direct numerical integration of the posterior distribution, we obtained the variance from 10^5 realizations of the numerical estimation for various values of exponent α and the numbers of neurons N (colored circle points). $M = 10$. Dashed lines are the theoretical prediction corresponding to the finite size Fisher information Eq. (S15), and the solid line is the one in the limit of large numbers of neurons ($N \to \infty$) that is given Eq. (S16).

where, p is the probability density function given by Eq. (S1). We prepare a set of M realizations of the activity of N neurons based on Eq. (S2), then put them into the log-likelihood function Eq. (S51) and numerically integrate probability distributions Eq. (S1) for values of θ to find the maximum likelihood estimate for the neural activity, i.e., the value of θ that maximizes the log-likelihood function (Fig. S2). By repeating the procedure, we compute the inverse of the variance of the estimates, which must asymptotically converge to the Fisher information in the limit of large numbers of observations $M \to \infty$ due to the Cramer-Rao theorem.

Figure S3 shows the numerically obtained inverse of the variance of the estimation error as functions of the power-law exponent α for various values of N. Each dotted line corresponds to the derived Fisher information, Eq. (S15) in the main text. One can confirm that numerical results well agree with the analytical predictions, and they monotonically converge to the solid line representing the Fisher information in the limit of large number of neurons Eq. (S16).

6. Discrepancy between analytical predictions and numerical results at very large noise strengths

In this section, we numerically investigate how the analytical predictions for Fisher information deviate from numerical results when noise strength increases to very large values. We perform the maximum likelihood estimation described in the previous section, but for very large values of the noise strengths σ_0 and σ_1. The results are shown in Figure S4. (To highlight the differences, the vertical axis in the bottom panels is presented on a logarithmic scale.) As expected, we observe that the discrepancy between the predictions and numerical results gradually increases as the neural noise strength σ_0 becomes larger. However, the analytical predictions still capture the qualitative behavior of the variance of the estimation error. We also found that the discrepancy appears to remain small when the input noise strength σ_1 increases. This may be because the variance of the estimation error is already too large for the difference to be recognized.

7. Fisher information for encoding neurons with an eigen power spectrum described by a broken power law

A recent study suggested that the eigen power spectrum is better described by a broken power law with two distinct exponents rather than a single power law with an exponent close to unity (1). Specifically, this study indicates that the first ten eigenvalues follow a shallow slope with an exponent $\alpha_1 \sim 0.5$, while the remaining eigenvalues decay more steeply with an exponent $\alpha_2 \sim 1.2$ (top panels of Figure S5). In this case, the zeta function in the analytical expression of the Fisher information needs to be replaced by the sum of two terms that represent the two slopes of the broken power law. More precisely, instead of

$$H_N(\alpha - 2/D) = \sum_{n=1}^{N} n^{-\alpha+2/D}, \qquad \text{[S52]}$$

we need to use

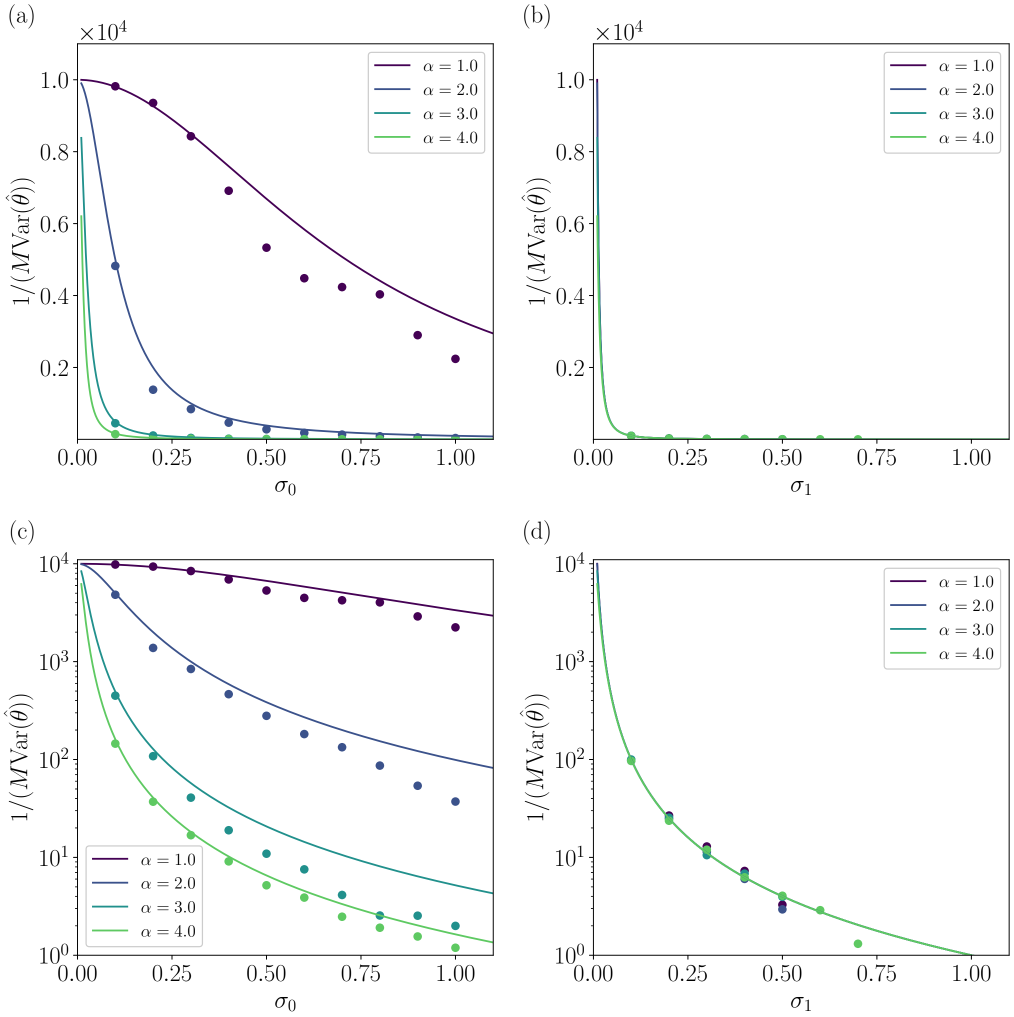

Fig. S4. The inverse of the variance of the estimation error for the maximum likelihood estimation of the input stimulus under large noise strengths. (a) The inverse of the variance as a function of neural noise strength σ_0 for various values of α, with input noise strength fixed at $\sigma_1 = 0.01$. (b) The same as (a), but as a function of input noise strength σ_1, with neural noise strength fixed at $\sigma_0 = 0.01$. (c) and (d) The same as (a) and (b), respectively, but with a logarithmic scale for the vertical axis.

$$H_N(\alpha_1 - 2/D, \alpha_1 - 2/D) = \sum_{n=1}^{10} n^{-\alpha_1 + 2/D} + 10^{\alpha_2 - \alpha_1} \sum_{n=11}^{N} n^{-\alpha_2 + 2/D}. \quad \text{[S53]}$$

Here, the factor $10^{\alpha_2 - \alpha_1}$ ensures the continuity of the broken power law at $n = 10$.

We plot this modified Fisher information in Figure S5 (bottom panels). The bottom left panel shows the original Fisher information in dependence of α, while the bottom middle and bottom right panels show the modified Fisher information as a function of the first slope α_1 and the second slope α_2, respectively. While the specific form of the Fisher information changes due to this modification, the qualitative properties remain consistent. For example, the information does not decrease as the exponents decrease, regardless of whether a single or broken power law is assumed.

One may notice that the decrease in Fisher information for the broken power law is relatively small when the second exponent α_2 increases (the bottom right panel), particularly for $D = 1$ (inset of the panel). This is due to the first segment of the broken power law. To understand this, consider the Fisher information of the original unbroken power law. As the exponent α increases, the slope of the power law becomes steeper, and the effective dimensionality of neural coding is finally reduced to one. Mathematically, this corresponds all terms in $H_N(\alpha)$, except the first, vanish in the limit as $\alpha \to \infty$, leading to a decrease in Fisher information via Eq. (S42). A similar reduction in dimensionality occurs for the broken power law when the first exponent α_1 increases, as all terms in Eq. (S53), except for the term with $n = 1$, converge to zero as $\alpha_1 \to \infty$. However, the first segment of the broken power law is independent of the second exponent α_2 (the top left panel in Figure S5). Thus, while increasing α_2 does reduce dimensionality, the first 10 dimensions, corresponding to the first sum in Eq. (S53), remain

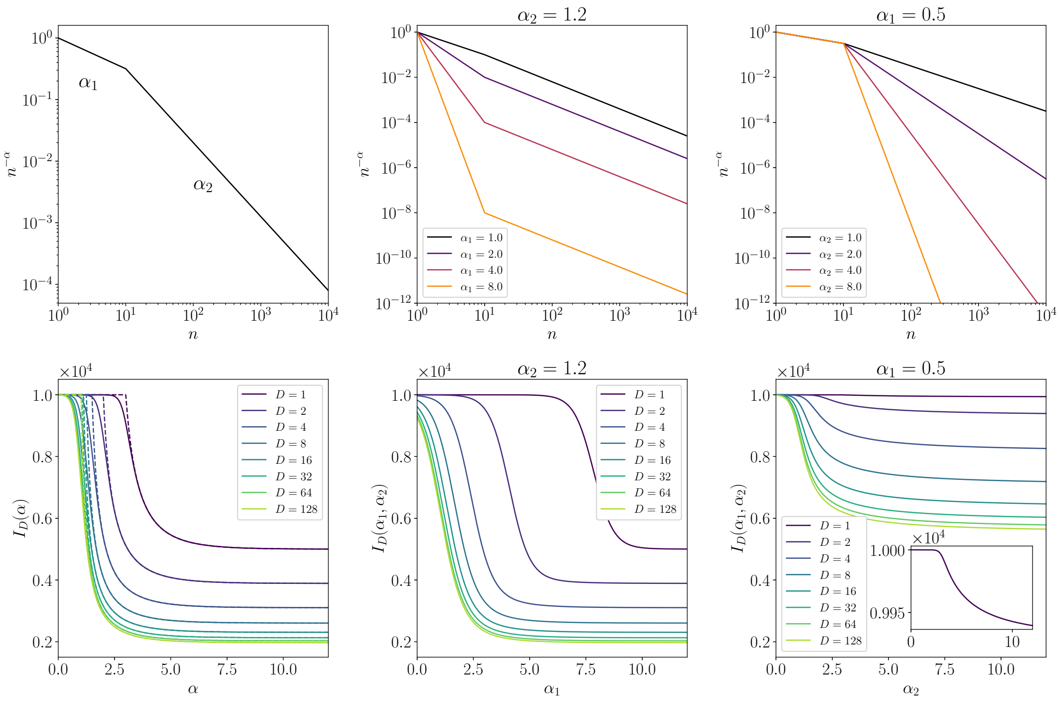

Fig. S5. Broken power law for the eigen power spectrum and the corresponding Fisher information. (Top left) Eigen power spectrum following a broken power law: the exponent of the first segment for $n \leq 10$ is $\alpha_1 = 0.5$, while that of the second segment for $n > 10$ is $\alpha_2 = 1.2$. (Top middle and top right) Eigen power spectra for different values of α_1 (top middle) and α_2 (top right). (Bottom left) The original Fisher information as a function of α for different input dimensions D. (Bottom middle) The modified Fisher information as a function of α_1. (Bottom right) The modified Fisher information as a function of α_2. The inset in this panel shows the result for $D = 1$.

finite even as $\alpha_2 \to \infty$. This weakens the decrease in H_N and the Fisher information. The effect is more pronounced for $D = 1$, as the remaining terms, represented by the first sum in Eq. (S53), are larger for smaller values of D. Whether this dependence on the exponents of the broken power law has some implications for neural population coding remains an interesting topic for future study.

References

1. DA Pospisil, JW Pillow, Revisiting the high-dimensional geometry of population responses in visual cortex. *bioRxiv* (2024).
